# Precise, Orthogonal Remote-Control of Cell-Free Systems Using Photocaged Nucleic Acids

**DOI:** 10.1021/jacs.3c01238

**Published:** 2023-04-19

**Authors:** Giacomo Mazzotti, Denis Hartmann, Michael J. Booth

**Affiliations:** † Department of Chemistry, University of Oxford, Mansfield Road, OX1 3TA Oxford, U.K.; ‡ Department of Chemistry, University College London, 20 Gordon Street, WC1H 0AJ London, U.K.

## Abstract

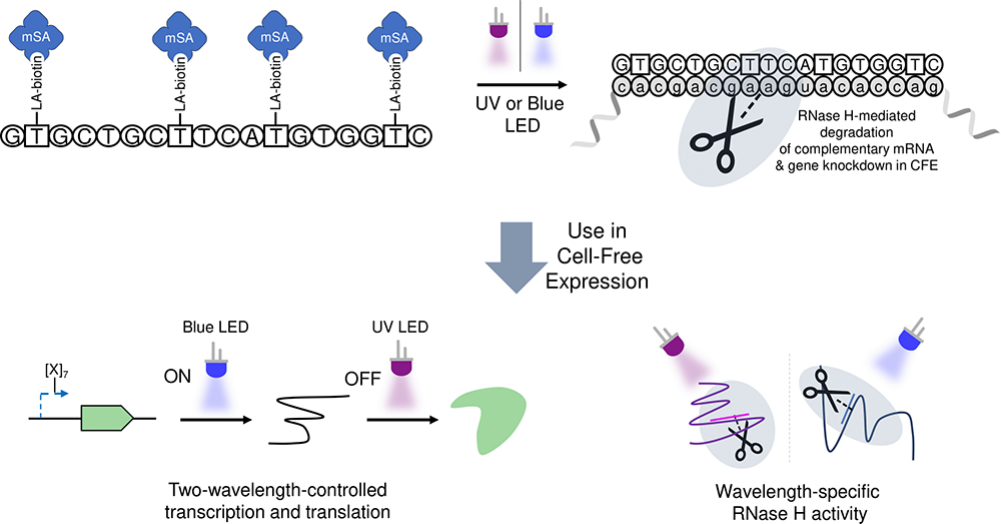

Cell-free expression of a gene to protein has become a vital tool in nanotechnology and synthetic biology. Remote-control of cell-free systems with multiple, orthogonal wavelengths of light would enable precise, noninvasive modulation, opening many new applications in biology and medicine. While there has been success in developing ON switches, the development of OFF switches has been lacking. Here, we have developed orthogonally light-controlled cell-free expression OFF switches by attaching nitrobenzyl and coumarin photocages to antisense oligonucleotides. These light-controlled OFF switches can be made from commercially available oligonucleotides and show a tight control of cell-free expression. Using this technology, we have demonstrated orthogonal degradation of two different mRNAs, depending on the wavelength used. By combining with our previously generated blue-light-activated DNA template ON switch, we were able to start transcription with one wavelength of light and then halt the translation of the corresponding mRNA to protein with a different wavelength, at multiple timepoints. This precise, orthogonal ON and OFF remote-control of cell-free expression will be an important tool for the future of cell-free biology, especially for use with biological logic gates and synthetic cells.

## Introduction

Cell-free expression (CFE) is a highly versatile tool used to achieve rapid RNA/protein synthesis through *in vitro* transcription and translation (IVTT) of natural or synthetic DNA.^1^ Avoiding the limitations of cell-based synthetic biology, such as laborious genetic encoding, gene delivery, and slow design-build-test cycles,^2^ CFE is ideal for high-throughput drug screening,^3^ the study of biological processes,^4−6^ gene circuits,^7^ and the purification of proteins otherwise challenging to express (e.g., containing non-natural amino acids).^8,9^ As a result, CFE technologies have recently gained great interest in the biomanufacturing field and industry.^10^ Moreover, CFE mixtures can be encapsulated in lipid bilayers to form synthetic cells^11^ for applications in drug delivery^12^ and studying cellular communication.^13^

The ability to control the function of these cell-free systems will be a key step to advance this technology toward more complex gene circuits or *in situ* formation and release of therapeutics.^14,15^ Control can be achieved with molecular inputs for RNA switches^16,17^ and transcription factor-based biosensors,^18,19^ with the analyte being the trigger for CFE activation. However, these signal molecules can be difficult to apply as, when added at different timepoints, they will change the concentrations of the components and are mostly not applicable for encapsulated systems such as synthetic cells. Furthermore, most of these molecular systems have been optimized for use in living cells and do not show optimal activity in cell-free systems. On the other hand, light is an ideal stimulus for controlling cell-free system systems as it can be applied remotely to closed systems in a spatiotemporal manner, has low toxicity, and is bioorthogonal to most biological processes.^15,20^ Furthermore, unlike other remote stimuli, it is possible to use multiple, orthogonal wavelengths of light to control different biological processes in the same system, opening up the prospect of precise, remote-control of cell-free systems.

Gene expression in cell-free systems has previously been controlled with light,^20^ either by chemically introducing light-activated photocages into the DNA templates for transcription^21,22^ or by using light-sensitive proteins.^23,24^ The majority of these systems have leaky “off-states,” where expression still occurs without light, and in the case of light-sensitive proteins require the expression of additional genes, both of which limit their application. We have previously developed an approach for the light activation of cell-free protein synthesis (CFPS) by attaching photocaged biotin/monovalent streptavidin (mSA) to a T7 promoter sequence to sterically block transcription, prior to illumination.^25^ Photocleavable biotin linkers were attached to amino-modified oligonucleotides and used as primers to amplify any gene of interest for control using light. The advantage of this approach is that it is made from commercially available modified nucleic acids and photocleavable biotin, and has a tight “off-state,” with negligible expression without illumination. We have used this light-activated DNA (LA-DNA) to spatiotemporally activate protein synthesis in synthetic tissues,^25,26^ to activate communication between synthetic cells and living cells,^27^ and, by employing orthogonal photocages, to engineer a dual-wavelength cell-free AND-gate.^28^

**Figure 1:**
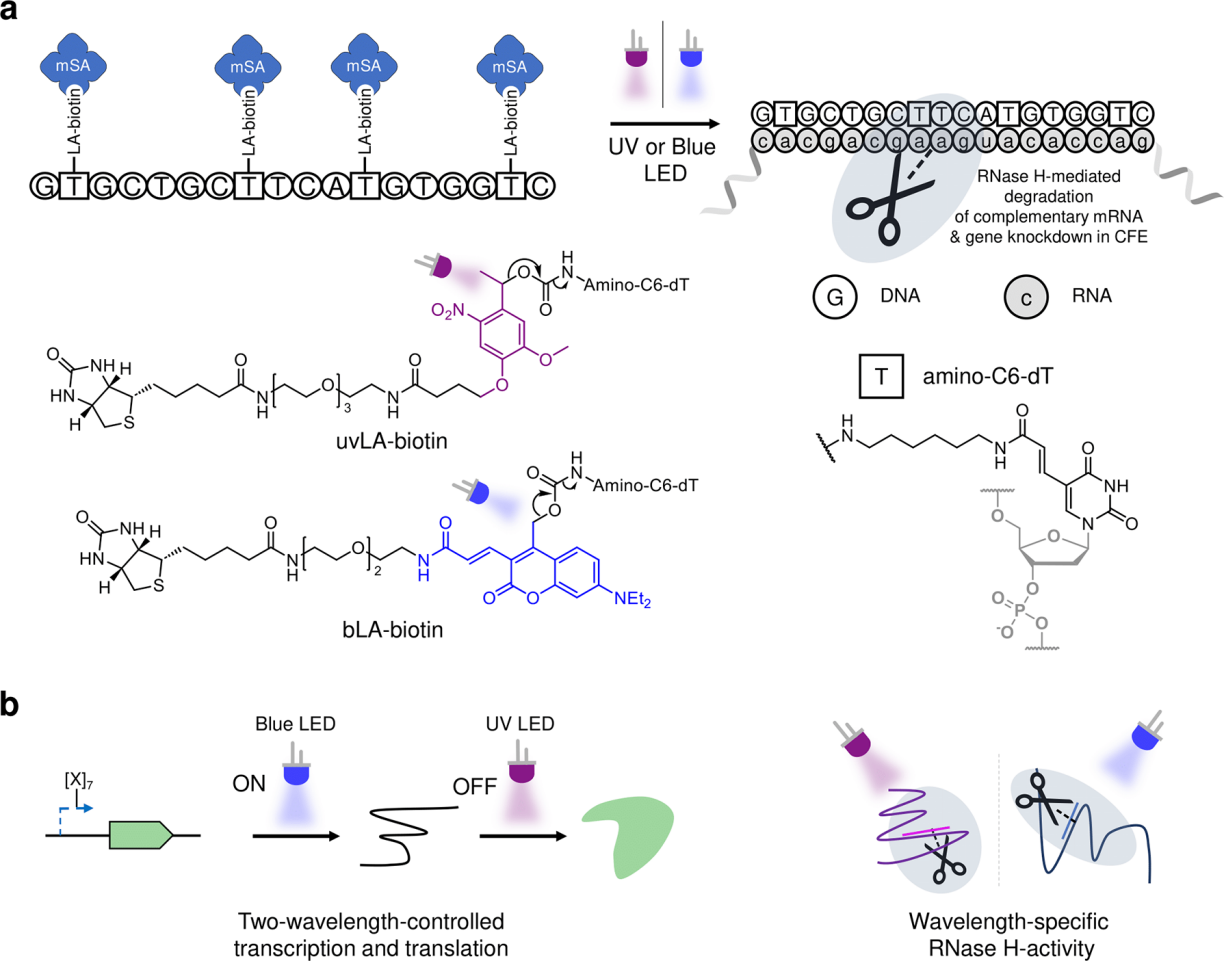
UV- and blue-light-activated antisense oligonucleotides (ASOs). (a) The ASOs were modified with light-activatable biotin moieties attached to monovalent streptavidin (mSA) to sterically prevent RNase H-mediated degradation. Light activation cleaves off the blocking groups restoring RNase H activity. Modification was achieved through commercially available DNA containing amino-C6-dT bases. (b) These light-activated ASOs were applied to orthogonally and precisely remote-control cell-free expression.

The main disadvantage of our current approach is that it is an irreversible ON switch. Azobenzene-modified DNA promoters have been used as photoreversible switches; however, they are leaky in the off-state and do not degrade the RNA already generated.^29^ In this paper, we have applied our simple photocleavable biotin/streptavidin approach to develop light-controlled CFPS OFF switches from antisense oligonucleotides (ASOs) (Figure 1). ASOs are short DNA sequences that can selectively degrade a target mRNA in the presence of Ribonuclease H (RNase H), a common endonuclease.^30^ While light-activated ASOs have previously been generated, they are difficult to synthesize and orthogonally controlled versions have never been realized.^31−33^ The use of ASOs in cell-free systems has been greatly underexplored; however, “transfection-style” methods have been developed to explore their use in synthetic cells.^34^ Here, we attached orthogonal UV (nitrobenzyl) and blue (coumarin) photocaged biotins (both of which have well-studied photocleavage mechanisms^35,36^), and then monovalent streptavidins, to amino-C6-dTs positioned across ASOs to tightly control gene knockdown in cell-free systems using light (Figure 1a). We demonstrated the orthogonal degradation of two different mRNAs, depending on the wavelength used, and combined UV-controlled ASOs with our previously generated blue-light-activated DNA templates to precisely remote-control CFPS (Figure 1b).

## Results and Discussion

Initially, we tested three variants of an ASO sequence we had previously identified that targets the mRNA for mVenus (mV), a yellow fluorescent protein.^37^ Each variant contained three thymines (dTs) replaced with amino-C6-dTs at different positions. The three amino-ASOs were reacted with a biotinylated 2-nitrobenzyl *N*-hydroxysuccinimide (NHS)-ester photocleavable group (uvLA-biotin, Figure 1) and purified by HPLC (see the Supporting Information), prior to binding either monovalent streptavidin (mSA) or wild-type streptavidin (tetSA) to sterically block RNase H from binding the hybrid DNA:RNA duplex. These UV-light-activatable (uvLA) ASOs were tested against mV-mRNA with or without illumination (Supporting Information, Figure S1) and analyzed by agarose gel electrophoresis. A better off-state (least mRNA degradation) was observed when the modified dTs were distributed throughout the entire sequence in ASO uvLA-**V1**, rather than concentrated at the terminus. Moreover, when bound to mSA rather than tetSA, the light-sensitive moieties appeared to photocleave more efficiently upon UV irradiation, resulting in a better ON/OFF ratio. Thus, we continued using mSA in the subsequent experiments.

**Figure 2:**
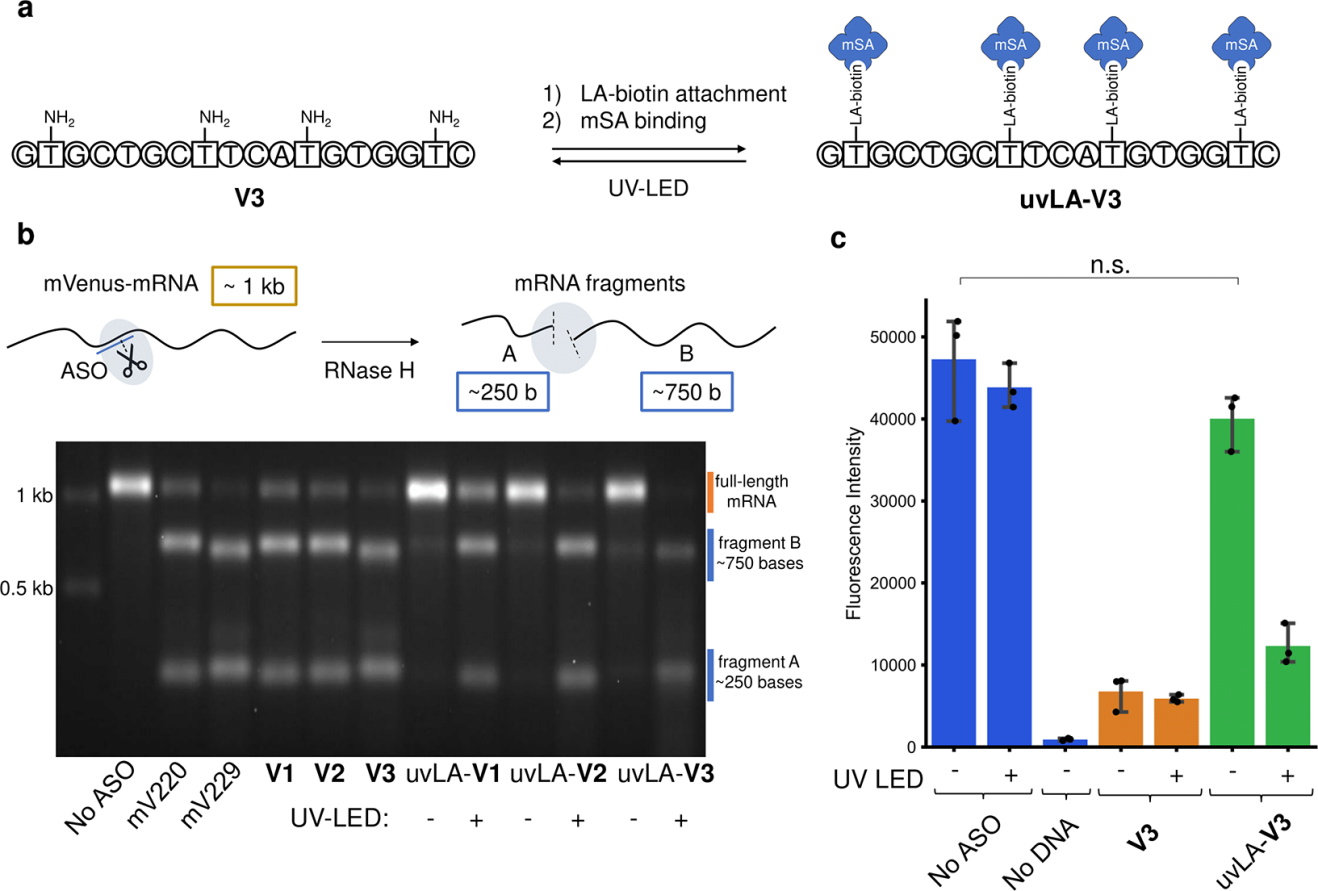
Light-activated antisense oligonucleotide (LA-ASO) construct formation and activity in RNase H-mediated mRNA degradation and cell-free protein synthesis (CFPS) reactions. (a) Formation of uvLA-**V3** ASO modified with four UV-photocleavable biotin groups and bound with monovalent streptavidin. (b) Testing of different ASO sequences with three and four modifications in an RNase H-mediated mVenus-mRNA degradation reaction. ASOs mV220 and mV229 are the unmodified sequences of **V2** and **V3**. See Supporting Information Table S4 for further details on the sequences. (c) Light-controlled knockdown of cell-free protein synthesis using uvLA-**V3**. Error bars represent the 95% confidence interval; ns (nonsignificant) = *p*-value > 0.05.

To produce a better ON/OFF ratio, we proceeded to substitute one further dT with amino-C6-dT, for a total of four modifications (Figure 2a). In addition, we screened for a new ASO targeting the same mRNA region, but with a higher potency and thymidines denser and more symmetrically distributed in the sequence (Figure 2b [mV220 vs mV229] and Supporting Information, Figure S2). Both the previous and the newly designed sequences containing four amino-C6-dTs (**V2** and **V3**, respectively) were then modified with uvLA-biotin and caged with mSA (Figure 2a). As expected, the addition of a fourth amino-dT resulted in a negligible decrease in ASO activity, while a fourth photocleavable streptavidin moiety visibly improved the “on/off-state” (Figure 2b).

As modified ASO uvLA-**V3** showed the best ON/OFF ratio, this was selected for further experiments. To control *in vitro* transcription (IVT), a linear DNA template encoding mVenus was transcribed in the presence of no ASO, **V3**, or uvLA-**V3**, and RNase H (Supporting Information, Figure S3). Without illumination, similar levels of mRNA were transcribed when using uvLA-**V3** as if no ASO was present, whereas when IVT took place following illumination, mRNA degradation was observed at similar levels of **V3** amino-ASO (representing 100% photocleavage). We then moved to control cell-free protein synthesis with the uvLA-**V3** ASO, using a commercial CFPS kit (PURExpress). **V3** or uvLA-**V3**, and the mV DNA template were added to the CFPS system (supplemented with RNase H), and the yield of intact mVenus was measured by fluorescence after 4 h (Figure 2c). UV light irradiation in the absence of an ASO only resulted in a minor, nonsignificant decrease (*p*-value = 0.23) in protein synthesis levels, showing little/no UV damage. The amino-ASO **V3** decreased mV synthesis by 89% compared to the no ASO control, with no significant difference following illumination (*p*-value = 0.265). In the absence of UV light, uvLA-**V3** only decreases protein synthesis by ∼15% (nonsignificant, *p*-value = 0.084), whereas when irradiated with UV for 5 min, protein synthesis was reduced by ∼75% compared to the no ASO control, and 84% of ASO activity was recovered compared to the amino-ASO **V3**. These results demonstrate that we were able to control gene knockdown of CFPS using our light-activated ASOs.

**Figure 3:**
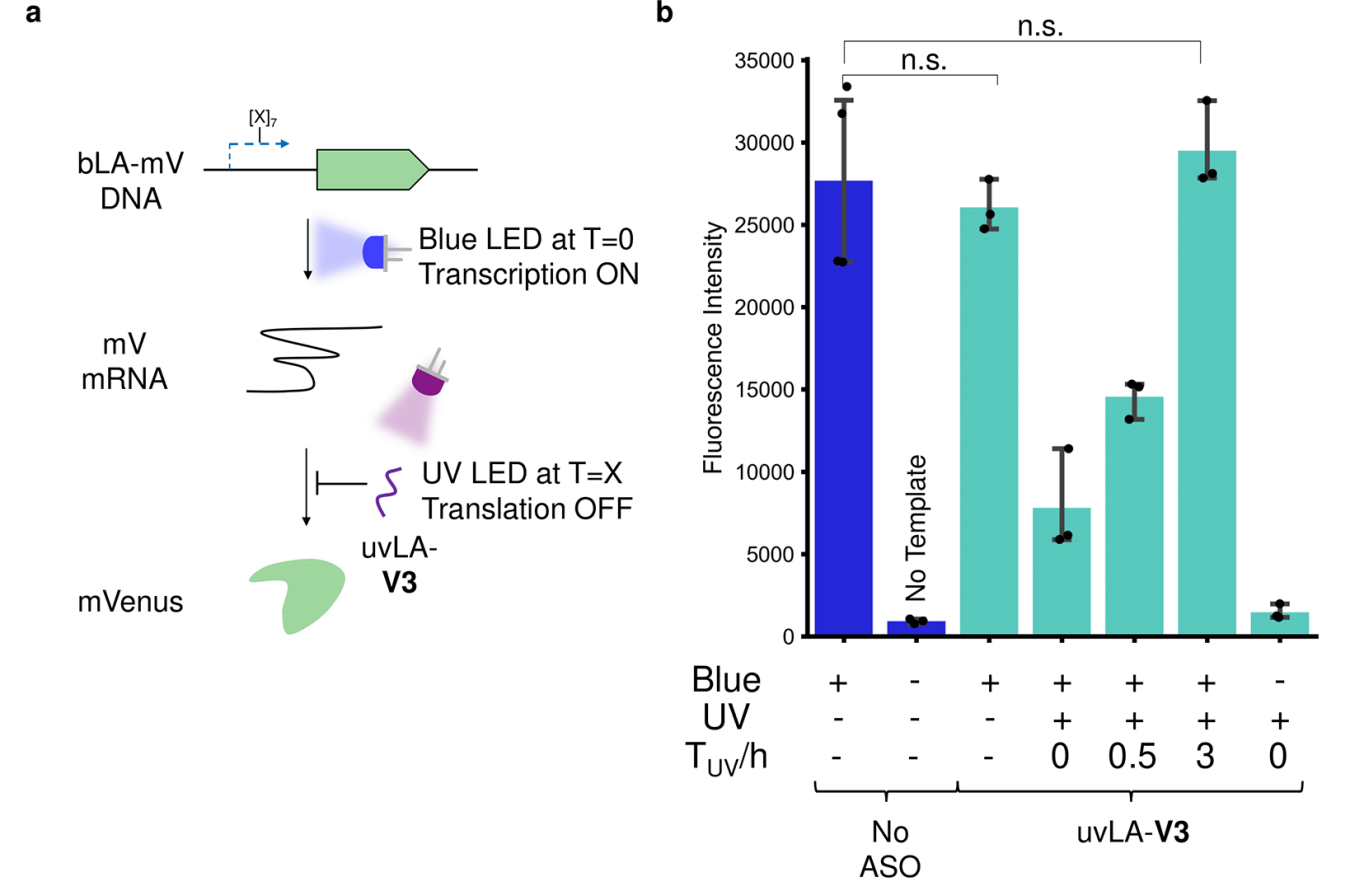
Two-wavelength activation and deactivation of cell-free protein synthesis. (a) Expression of mVenus is turned ON from a bLA-mV DNA template by irradiation with blue light. UV light can then activate the uvLA-**V3** ASO to turn the expression OFF again. (b) Control of cell-free protein synthesis by activation of transcription using blue light and deactivation of translation at different timepoints using UV, which triggers RNase H-mediated degradation of the mRNA. Error bars represent the 95% confidence interval; ns (nonsignificant) = *p*-value > 0.05.

We recently reported a blue-light-activatable (bLA) photocaging group to control CFPS, which acts orthogonally to UV light.^28^ By combining a blue-light-activatable (bLA) mV DNA template with the uvLA-**V3** ASO, we envisaged a system where transcription could be selectively turned ON with blue light and translation turned OFF with UV light (Figure 3a). The feasibility of this approach was initially indicated by the orthogonality of the UV–vis spectra of the previously prepared bLA-T7 promoter^28^ compared to the spectrum of the prepared uvLA-**V3** ASO (Supporting Information). We initially tested this in an IVT reaction using the bLA-mV DNA template in the presence of RNase H and uvLA-**V3** (Supporting Information, Figure S4). Following blue light irradiation, we observed an increasing amount of mV-mRNA produced over 3 h, as measured on agarose gel electrophoresis, similar to the amount produced from a unmodified DNA template (Supporting Information, Figure S5). The IVT reactions were then incubated for a further hour with or without UV illumination. When irradiated with UV light, the mRNA was almost completely degraded in 1 h, due to the activation of uvLA-**V3**. Without UV illumination, no degradation was observed, demonstrating uvLA-**V3** was not activated.

After the successful proof of concept with an IVT system, we moved on to apply this blue ON and UV OFF system in CFPS (Figure 3a). We incubated the bLA-mV DNA template in the CFPS system at 37 °C in the presence of uvLA-**V3** and RNase H for 4 h under different illumination conditions and measured intact protein yield by fluorescence (Figure 3b). Illumination with only blue light showed an equal protein output compared to the no ASO control (*p*-value = 0.196), due to uvLA-**V3** not activating under blue light. Irradiation with UV light alone did not activate the bLA-mV DNA template, and no protein was produced after 4 h, as only the ASO was activated. UV light was then applied at different timepoints, following blue light activation of the template, to halt cell-free protein synthesis upon demand by degrading the mRNA already generated. When irradiated with UV at the same time as blue light, prior to incubation, a high degree of gene knockdown (74%) was observed, as any mRNA formed would be quickly degraded by RNase H and the uncaged ASO. When irradiating with UV following blue light activation and 30 min incubation, protein synthesis had already initiated but was halted at 51%, compared to when no ASO was present. This is in line with previous data, showing that CFPS systems produce a substantial amount of mRNA and protein in the first stages of the reaction.^38^ Lastly, when irradiated with UV following blue light activation and 3 h incubation, no reduction in protein output was observed (*p*-value = 0.519), as expected, because mVenus production already reached a plateau. This demonstrated a two-wavelength ON and OFF switch for CFPS, with the ability to temporally activate and halt protein synthesis.

**Figure 4:**
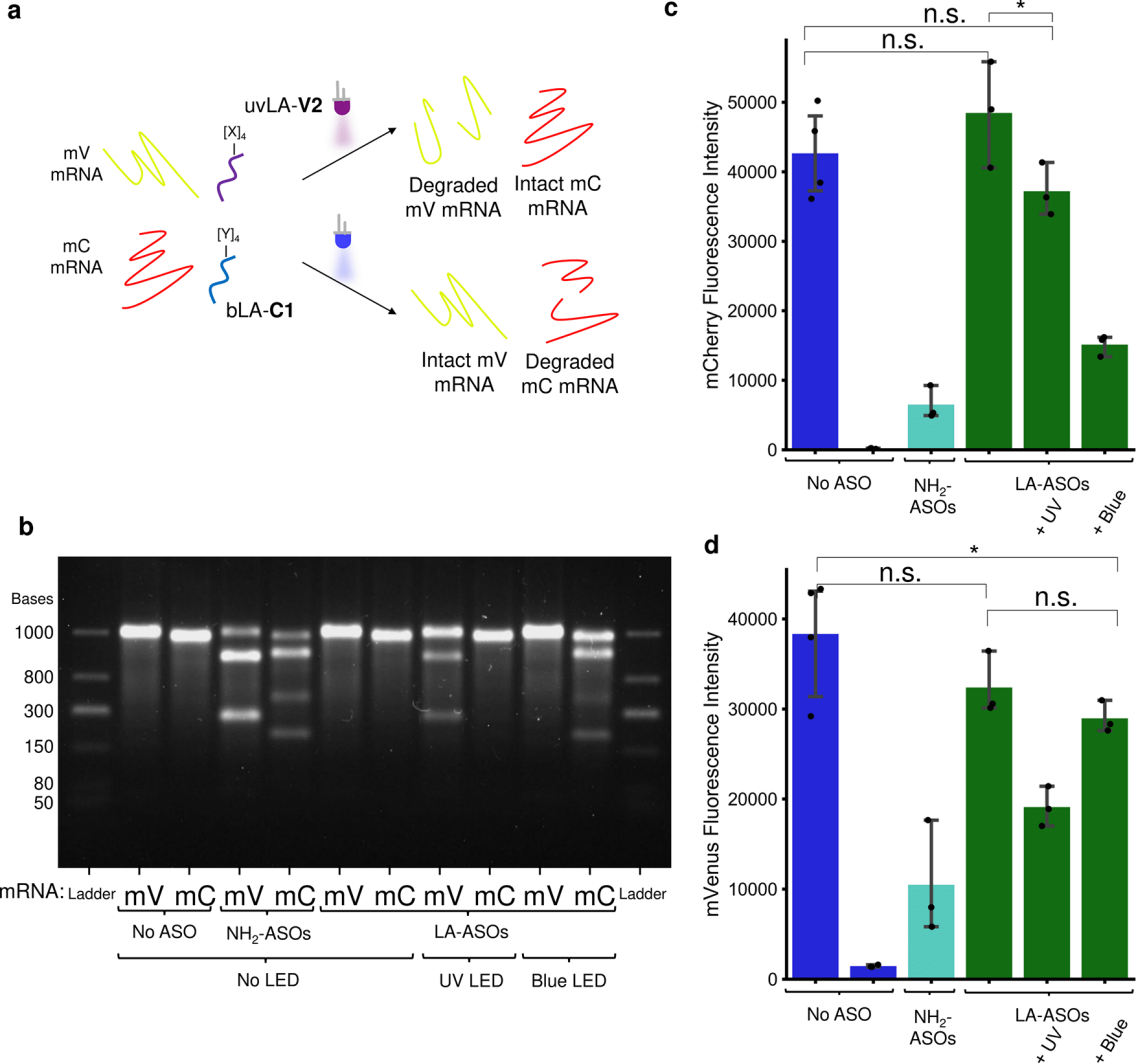
Two-wavelength control of gene knockdown. (a) The antisense oligonucleotides (ASOs) uvLA-**V2** and bLA-**C1** target mVenus (mV) and mCherry (mC), respectively, and are selectively activated by UV and blue light. The RNase H-mediated degradation of mRNA reaction (b) and cell-free protein synthesis assay in the presence of RNase H (c, d) show orthogonality between the ASOs and their activation with light. Error bars represent the 95% confidence interval; ns (nonsignificant) = *p*-value > 0.05, * = *p*-value < 0.05.

As we had orthogonal UV and blue photocages that could be attached to amino-C6-dT modifications, we next aimed to generate two ASOs that would bind different mRNA and selectively degrade their target with each wavelength (Figure 4a). To allow the fluorescent measurement of a different target in CFPS, we aimed to identify a new ASO sequence that could target the mRNA encoding for the red fluorescent protein mCherry (mC), without binding mV-mRNA. Being derived from fluorescent proteins of different organisms (dsRed vs. avGFP),^39,40^ mC has an orthogonal excitation/emission spectrum to mV and a large difference in the DNA sequence. After screening several ASOs to identify a good target point in mC-mRNA, with sequences containing multiple Ts across the length of the ASO (Supporting Information, Figure S6), a second screening process was carried out to find a sequence with orthogonality to mVenus (Supporting Information, Figure S7). The same test was also performed with the mVenus ASOs, in which we identified that **V2** was orthogonal to mC, whereas **V3** showed some crosstalk (Supporting Information, Figure S8).

The two ASOs chosen from these screens for orthogonal targeting of mC (**C1**) and mV (**V2**) both contained four amino-C6-dT modifications. They were modified with bLA-biotin and uvLA-biotin (Figure 1), respectively, purified by HPLC, validated for orthogonality by UV–vis (Supporting Information), and bound to mSA (forming bLA-**C1** and uvLA-**V2**). mV- and mC-mRNA were incubated with the two photocaged ASOs and RNase H, to test light-controlled mRNA degradation (Figure 4b). In the absence of irradiation, both mRNAs remained intact. Strikingly, upon illumination with blue light, mC-mRNA was degraded while mV-mRNA stayed intact. Similarly, mV-mRNA was degraded selectively after irradiation with UV light, with no degradation being observed for mC-mRNA. We then used these orthogonal LA-ASOs to selectively control the cell-free protein synthesis of the two different proteins (Figure 4c). The DNA templates of mV and mC, bLA-**C1**, uvLA-**V2**, and RNase H were added to the CFPS system, incubated for 4 h following different patterns of illumination, and the fluorescence of both proteins was measured. Without illumination, both proteins were expressed to similar levels observed in the absence of ASOs (17% increase for mC and 23% decrease for mV, both nonsignificant). Upon UV irradiation, uvLA-**V2** selectively knocked down mV fluorescence by 56%, whereas mC was only reduced by 16% (nonsignificant, *p*-value = 0.129). Vice versa, upon blue light irradiation, bLA-**C1** selectively knocked down mC fluorescence by 62%, with a 30% reduction observed for mV fluorescence (significant against positive control, *p*-value = 0.032). Excitingly, this demonstrated we were able to control gene knockdown in CFPS using two orthogonally light-controlled ASOs.

## Conclusions

Here, we have demonstrated the synthesis and application of light-controllable ASOs for the precise, remote-control of cell-free systems. These ASOs were easily made from commercially available oligonucleotides and two orthogonal color photocages, and were able to tightly control protein output in CFPS using light. We were able to combine these light-controlled ASOs with our previously developed light-activatable DNA templates, to activate transcription with one wavelength of light and halt translation with a second wavelength. Lastly, we generated two light-controlled ASOs that, for the first time could target different mRNAs orthogonally, for selective two-wavelength gene knockdown in CFPS. Being able to remotely and orthogonally activate and deactivate gene expression on demand in cell-free systems will open new possibilities for designs of gene circuits and synthetic cells, and lead to new applications in synthetic biology.

## Supporting Information Available

DNA/RNA sequences; materials and methods; screening of ASOs; gel analysis of ASO orthogonality; and characterization data (PDF)
