# Supplementary material for: Precise, Orthogonal Remote-Control of Cell-Free Systems Using Photocaged Nucleic Acids
Source: J Am Chem Soc. 2023 Apr 19;145(17):9481–7. doi: 10.1021/jacs.3c01238 (PMC10161223; doi:10.1021/jacs.3c01238)
Supplement: Supplementary file 1 — ja3c01238_si_001.pdf [file ja3c01238_si_001.pdf]

Supplementary information

Precise, orthogonal remote-control of cell-free systems using photocaged nucleic acids

Giacomo Mazzotti[1‡], Denis Hartmann[1‡] and Michael J. Booth[1,2*]

[1]Department of Chemistry, University of Oxford, Mansfield Road, OX1 3TA, Oxford, UK.

[2]Department of Chemistry, University College London, 20 Gordon Street, WC1H 0AJ, London UK.

[‡]These authors contributed equally.

[*]Correspondence: m.j.booth@ucl.ac.uk

Table of Contents

DNA and mRNA sequences 4

DNA sequences 4

mRNA sequences 5

Oligonucleotide Sequences 6

Sequences of oligonucleotides used in PCR 6

Antisense oligonucleotides sequences 6

Materials and Methods 8

General 8

Formation of biotinylated UV-photocleavable antisense oligonucleotides 8

Preparation of blue light-activatable antisense oligonucleotides 8

HPLC purification of modified antisense oligonucleotides 8

Binding of streptavidin 8

Preparation of linear template DNA 8

Transcription of mVenus and mCherry mRNA 9

RNase H-mediated mRNA degradation 9

UV irradiation conditions 9

Blue light irradiation conditions 9

In vitro transcription control using uvLA-V3 9

Two-wavelength control of in vitro transcription 10

Light-controlled gene knockdown in cell-free protein synthesis with uvLA-V3 10

Control of transcription and gene knockdown with two wavelengths in cell-free protein synthesis 10

Two-wavelength control of RNase H-mediated mRNA degradation 11

Two wavelength-controlled gene knockdown in cell-free protein synthesis 11

Agarose gel electrophoresis 11

RNase H-mediated degradation of mRNA experiments 12

RNase H-mediated mVenus-mRNA degradation controlled by UV 12

RNase H-mediated mVenus-mRNA degradation with different unmodified ASO sequences 13

Control of in vitro transcription experiments 14

In vitro transcription of mVenus-DNA controlled with UV light 14

Two-wavelength control of in vitro transcription of mVenus 15

Control of mVenus transcription with amino-ASO 16
Initial screening of antisense oligonucleotides targeting mCherry 17
Evaluation of mCherry-ASOs crosstalk to mVenus-mRNA 18
Orthogonality between mCherry- and mVenus-ASOs......................... 19
HPLC purification of biotinylated antisense oligonucleotides 20
Preparation of uvLA-V1 biotin 20
Preparation of uvLA-V2 biotin 21
Preparation of uvLA-V3 biotin 22
Preparation of bLA-C1 biotin 23
UV-Vis Traces of LA-ASOs 24
Oligonucleotide Mass Spectrometry 25
LC-MS spectra of uvLA-V1 biotin 26
LC-MS spectra of uvLA-V2 biotin 28
LC-MS spectra of uvLA-V3 biotin 30
LC-MS Spectra of bLA-C1 biotin 32

DNA and mRNA sequences

DNA sequences

Gene	Sequence (5' → 3')
mVenus	GAAATTAATACGACTCACTATAGGGTCTAGAAATAATTTTGTTTAACTTTAAGAAGGAGG<br>TATACAT**ATG**GTGAGCAAGGGCGAGGAGCTGTTCACCGGGGTGGTGCCCATCCTGGT<br>CGAGCTGGACGGCGACGTAAACGGCCACAAGTTCAGCGTGTCCGGCGAGGGCGAGG<br>GCGATGCCACCTACGGCAAGCTGACCCTGAAGCTCATCTGCACCACCGGCAAGCTGC<br>CCGTGCCCTGGCCCACCCTCGTGACCACCCTCGGCTACGGCCTGCAGTGCTTCGCCC<br>GCTACCCCGACCACATGAAGCAGCACGACTTCTTCAAGTCCGCCATGCCCGAAGGCTA<br>CGTCCAGGAGCGCACCATCTTCTTCAAGGACGACGGCAACTACAAGACCCGCGCCGA<br>GGTGAAGTTCGAGGGCGACACCCTGGTGAACCGCATCGAGCTGAAGGGCATCGACTT<br>CAAGGAGGACGGCAACATCCTGGGGCACAAGCTGGAGTACAACTACAACAGCCACAA<br>CGTCTATATCACCGCCGACAAGCAGAAGAACGGCATCAAGGCCAACTTCAAGATCCGC<br>CACAACATCGAGGACGGCGGCGTGCAGCTCGCCGACCACTACCAGCAGAACACCCCC<br>ATCGGCGACGGCCCCGTGCTGCTGCCCGACAACCACTACCTGAGCTACCAGTCCAAG<br>CTGAGCAAAGACCCCAACGAGAAGCGCGATCACATGGTCCTGCTGGAGTTCGTGACC<br>GCCGCCGGGATCACTCTCGGCATGGACGAGCTGTACAAG**TAA**TGAGGATCCCGGGAA<br>TTCTCGAGTAAGGTTAACCTGCAGGAGGCCTTTAATTAAGGTGGTGCGGCCGCGCTAG<br>CGGTCCCGGGGGATCGATCCGGCTGCTAACAAAGCCCGAAAGGAAGCTGAGTTGGCT<br>GCTGCCACCGCTGAGCAATAACTAGCATAACCCCTTGGGGCCTCTAAACGGGTCTTGA<br>GGGGTTTTTTGCTGAAAGGAGGAACTATATC
mCherry	GAAATTAATACGACTCACTATAGGGTCTAGAAATAATTTTGTTTAACTTTAAGAAGGAGG<br>TATACAT**ATG**GTGAGCAAGGGCGAGGAGGATAACATGGCCATCATCAAGGAGTTCATG<br>CGCTTCAAGGTGCACATGGAGGGCTCCGTGAACGGCCACGAGTTCGAGATCGAGGGC<br>GAGGGCGAGGGCCGCCCCTACGAGGGCACCCAGACCGCCAAGCTGAAGGTGACCAA<br>GGGTGGCCCCCTGCCCTTCGCCTGGGACATCCTGTCCCCTCAGTTCATGTACGGCTC<br>CAAGGCCTACGTGAAGCACCCCGCCGACATCCCCGACTACTTGAAGCTGTCCTTCCCC<br>GAGGGCTTCAAGTGGGAGCGCGTGATGAACTTCGAGGACGGCGGCGTGGTGACCGT<br>GACCCAGGACTCCTCCCTGCAGGACGGCGAGTTCATCTACAAGGTGAAGCTGCGCGG<br>CACCAACTTCCCCTCCGACGGCCCCGTAATGCAGAAGAAGACCATGGGCTGGGAGGC<br>CTCCTCCGAGCGGATGTACCCCGAGGACGGCGCCCTGAAGGGCGAGATCAAGCAGA<br>GGCTGAAGCTGAAGGACGGCGGCCACTACGACGCTGAGGTCAAGACCACCTACAAGG<br>CCAAGAAGCCCGTGCAGCTGCCCGGCGCCTACAACGTCAACATCAAGTTGGACATCA<br>CCTCCCACAACGAGGACTACACCATCGTGGAACAGTACGAACGCGCCGAGGGCCGCC<br>ACTCCACCGGCGGCATGGACGAGCTGTACAAG**TAA**TGAGGATCCCGGGAATTCTCGA<br>GTAAGGTTAACCTGCAGGAGGCCTTTAATTAAGGTGGTGCGGCCGCGCTAGCGGTCC<br>CGGGGGATCGATCCGGCTGCTAACAAAGCCCGAAAGGAAGCTGAGTTGGCTGCTGCC<br>ACCGCTGAGCAATAACTAGCATAACCCCTTGGGGCCTCTAAACGGGTCTTGAGGGGTT<br>TTTTGCTGAAAGGAGGAACTATATC

Supplementary Table 1: Sequences of linear DNA templates used for cell-free expression, produced by PCR. The T7-promoter and T7-terminator regions are underlined. Protein start and stop codons are in bold.

mRNA sequences

Gene	Sequence (5' → 3')
mVenus	GGGUCUAGAAAUAAUUUUGUUUAACUUUAAGAAGGAGGUAUACAU**AUG**GUGAGCAA<br>GGGCGAGGAGCUGUUCACCGGGGUGGUGCCCAUCCUGGUCGAGCUGGACGGCGAC<br>GUAAACGGCCACAAGUUCAGCGUGUCCGGCGAGGGCGAGGGCGAUGCCACCUACG<br>GCAAGCUGACCCUGAAGCUCAUCUGCACCACCGGCAAGCUGCCCGUGCCCUGGCCC<br>ACCCUCGUGACCACCCUCGGCUACGGCCUGCAGUGCUUCGCCCGCUACCCCGACCA<br>CAUGAAGCAGCACGACUUCUUCAAGUCCGCCAUGCCCGAAGGCUACGUCCAGGAGC<br>GCACCAUCUUCUUCAAGGACGACGGCAACUACAAGACCCGCGCCGAGGUGAAGUUC<br>GAGGGCGACACCCUGGUGAACCGCAUCGAGCUGAAGGGCAUCGACUUCAAGGAGGA<br>CGGCAACAUCCUGGGGCACAAGCUGGAGUACAACUACAACAGCCACAACGUCUAUA<br>UCACCGCCGACAAGCAGAAGAACGGCAUCAAGGCCAACUUCAAGAUCCGCCACAACA<br>UCGAGGACGGCGGCGUGCAGCUCGCCGACCACUACCAGCAGAACACCCCCAUCGGC<br>GACGGCCCCGUGCUGCUGCCCGACAACCACUACCUGAGCUACCAGUCCAAGCUGAG<br>CAAAGACCCCAACGAGAAGCGCGAUCACAUGGUCCUGCUGGAGUUCGUGACCGCCG<br>CCGGGAUCACUCUCGGCAUGGACGAGCUGUACAAGUAAUGAGGAUCCCGGGAAUUC<br>UCGAGUAAGGUUAACCUGCAGGAGGCCUUUAAUUAAGGUGGUGCGGCCGCGCUAG<br>CGGUCCCGGGGGAUCGAUCCGGCUGCUAACAAAGCCCGAAAGGAAGCUGAGUUGG<br>CUGCUGCCACCGCUGAGCAAUAACUAGCAUAACCCCUUGGGGCCUCUAAACGGGUC<br>UUGAGGGGUUUUUUG
mCherry	GGGUCUAGAAAUAAUUUUGUUUAACUUUAAGAAGGAGGUAUACAU**AUG**GUGAGCAA<br>GGGCGAGGAGGAUAACAUGGCCAUCAUCAAGGAGUUCAUGCGCUUCAAGGUGCACA<br>UGGAGGGCUCCGUGAACGGCCACGAGUUCGAGAUCGAGGGCGAGGGCGAGGGCCG<br>CCCCUACGAGGGCACCCAGACCGCCAAGCUGAAGGUGACCAAGGGUGGCCCCCUGC<br>CCUUCGCCUGGGACAUCCUGUCCCCUCAGUUCAUGUACGGCUCCAAGGCCUACGUG<br>AAGCACCCCGCCGACAUCCCCGACUACUUGAAGCUGUCCUUCCCCGAGGGCUUCAA<br>GUGGGAGCGCGUGAUGAACUUCGAGGACGGCGGCGUGGUGACCGUGACCCAGGAC<br>UCCUCCCUGCAGGACGGCGAGUUCAUCUACAAGGUGAAGCUGCGCGGCACCAACUU<br>CCCCUCCGACGGCCCCGUAAUGCAGAAGAAGACCAUGGGCUGGGAGGCCUCCUCC<br>GAGCGGAUGUACCCCGAGGACGGCGCCCUGAAGGGCGAGAUCAAGCAGAGGCUGA<br>AGCUGAAGGACGGCGGCCACUACGACGCUGAGGUCAAGACCACCUACAAGGCCAAG<br>AAGCCCGUGCAGCUGCCCGGCGCCUACAACGUCAACAUCAAGUUGGACAUCACCUC<br>CCACAACGAGGACUACACCAUCGUGGAACAGUACGAACGCGCCGAGGGCCGCCACU<br>CCACCGGCGGCAUGGACGAGCUGUACAAGUAAUGAGGAUCCCGGGAAUUCUCGAGU<br>AAGGUUAACCUGCAGGAGGCCUUUAAUUAAGGUGGUGCGGCCGCGCUAGCGGUCC<br>CGGGGGAUCGAUCCGGCUGCUAACAAAGCCCGAAAGGAAGCUGAGUUGGCUGCUG<br>CCACCGCUGAGCAAUAACUAGCAUAACCCCUUGGGGCCUCUAAACGGGUCUUGAGG<br>GGUUUUUUG

Supplementary Table 2: Sequences of mRNA used for RNase H-mediated mRNA degradation reactions and in vitro transcription experiments. In bold, the AUG start codon, where A was used as mRNA first position for antisense oligonucleotides numbering (see **Supplementary Table 4**).

Oligonucleotide Sequences

Unmodified oligonucleotides were purchased from IDT, and amino-modified oligonucleotides were purchased from ATDBio. All oligonucleotides were made up to 100 μM in 10 mM tris-EDTA buffer, pH 8.

Sequences of oligonucleotides used in PCR

Name	Sequence (5’ → 3’)
T7 Forward primer	GAAATTAATACGACTCACTATAGGGTCTAG
Reverse primer	GATATAGTTCCTCCTTTCAG

Supplementary Table 3: Sequence of primers used to generate the DNA templates shown in Supplementary Table S1 by PCR.

Antisense oligonucleotides sequences

Entry	Target	Nucleotide position on mRNA sequence	Length in number of bases	Number of amines	Sequence (5’ → 3’)	
1	mV	220	20	0	TTCATGTGGTCGGGGTAGCG	
2	mV	220	20	3	TXCATGTGGXCGGGGXAGCG	→ **V1**
3	mV	220	20	3	2XCAXGTGGTCGGGGTAGCG	→ **V1b**
4	mV	220	20	3	2XCAXGTGGXCGGGGTAGCG	→ **V1c**
5	mV	220	20	4	TXCATGXGGXCGGGGXAGCG	→ **V2**
6	mV	229	18	0	GTGCTGCTTCATGTGGTC	
7	mV	229	18	4	GXGCTGCXTCAXGTGGXC	→ **V3**
8	mV	224	16	0	TTCATGTGGTCGGGGT	
9	mV	229	17	0	TGCTGCTTCATGTGGTC	
10	mV	230	16	0	TGCTGCTTCATGTGGT	
11	mC	27	19	0	CTCCTTGATGATGGCCATG	
12	mC	81	19	0	CTCGAACTCGTGGCCGTTC	
13	mC	139	18	0	CTTCAGCTTGGCGGTCTG	
14	mC	147	19	0	CTTGGTCACCTTCAGCTTG	
15	mC	222	16	0	CTTCACGTAGGCCTTG	
16	mC	303	16	0	GTCCTCGAAGTTCATC	
17	mC	141	16	0	CTTCAGCTTGGCGGTC	
18	mC	139	16	0	TCAGCTTGGCGGTCTG	
19	mC	139	16	4	XCAGCTXGGCGGXCXG	→ **C1**
20	mC	140	15	0	TCAGCTTGGCGGTCT	
21	mC	135	22	0	CTTCAGCTTGGCGGTCTGGGTG	
22	mC	139	23	0	GTCACCTTCAGCTTGGCGGTCTG	
23	mC	141	21	0	GTCACCTTCAGCTTGGCGGTC	
24	mC	142	20	0	GTCACCTTCAGCTTGGCGGT	

Supplementary Table 4: List of antisense oligonucleotides (ASOs) used in this work. The table lists the mRNA target sequence, the starting position of ASO base

pairing with mRNA (counting from mRNA start codon, see **Supplementary Table 2**), the oligonucleotide length in number of bases, the number of amino modifications on the ASO and the sequence. The names assigned to specific sequences used throughout the text is indicated on the right. X = internal C6-hexylamino-dT modification, 2 = 5'-C6-hexylamino-phosphate-dT modification.

Materials and Methods

General
Experiments containing blue light-activatable biotin groups were performed under reduced laboratory lighting conditions (direct overhead lights turned off). Data was plotted using python's matplotlib and seaborn libraries. Error bars and confidence intervals were computed using seaborn and show a 95% confidence interval. T-tests were performed using python's scipy library, using the stats.ttest_ind() method and a one-tailed t-test was applied. Activation percentages were calculated using the following equation: $\frac{\mu_S-\mu_N}{\mu_R-\mu_N}\times 100$, where μ = mean, N = background sample, S = sample of interest, R = reference sample. Gel electrophoresis data was analysed using ImageLab software.

Formation of biotinylated UV-photocleavable antisense oligonucleotides
Amino-modified oligonucleotides (20 μM, ATDBio) (**Supplementary Table 4**) were added to a 0.5 mL DNA LoBind tube (Eppendorf) and reacted with PC Biotin-NHS ester (5 mM, Click Chemistry Tools) with 100 mM $NaHCO_3$, in a total volume of 50 μL (50% water 50% DMF). Reactions were incubated in the dark at room temperature for 1 hour, with frequent mixing and centrifugation, then left at 4 °C overnight. Reactions were quenched with 350 μL of 20 mM tris pH 7. The mixtures were then partially desalted by centrifugation with Amicon Ultra-0.5 mL centrifugal filters (Merck) according to the manufacturer's protocol, then purified further by HPLC.

Preparation of blue light-activatable antisense oligonucleotides
The amine-modified oligonucleotide (Entry 19, **Supplementary Table 4,** 23.5 μM) was added to a 1.5 mL DNA LoBind Tube (Eppendorf) and reacted with bLA-Biotin PFP Carbonate (as prepared previously[1], 17.5 mM) with MOPS pH 8.5 (119 mM) in 59% DMF in a total reaction volume of 34 μL. The reaction was incubated in a Thermomixer (Eppendorf) at 37 °C and 800 RPM overnight. The solution was diluted to 50 μL with H_2O, washed with $CHCl_3$ (3 x 200 μL), 50 μL DMSO added, transferred to a HPLC injection vial and purified by HPLC.

HPLC purification of modified antisense oligonucleotides
The modified oligonucleotides were purified by HPLC on an Agilent Polaris C-18 column (150 x 4.5 mm), heated to 50 °C, using a gradient of 5-28% MeCN over 36 minutes with 10 mM TEAB pH 8.5 throughout. The resulting oligonucleotides were lyophilised, resuspended in H_2O and analysed by LCMS for purity (details on LCMS below).

Binding of streptavidin
The biotinylated antisense oligonucleotides were incubated with monovalent streptavidin (kindly provided by Howarth's laboratory, Department of Biochemistry, University of Oxford) or tetravalent streptavidin (NEB) in a 4-fold excess to the number of photocleavable biotin groups. The reactions were incubated in the dark for 2-3 hours at room temperature, then left at 4 °C overnight.

Preparation of linear template DNA
PCR reactions were carried out using DreamTaq DNA polymerase MasterMix (2X, ThermoFisher), forward and reverse primers (**Supplementary Table 3**) at 0.25 μM

concentration and 0.04 ng/µL of the Hind III-digested plasmid as template, in a total reaction volume of 25 µL. The PCR was carried out according to the manufacturer's protocol for 35 cycles with an annealing temperature of 52 °C for 30 seconds, an extension time of 72 °C for 1 minute 15 seconds/kbp and a final extension at 72 °C for 10 minutes. The resulting DNA was then purified using the GeneJet PCR purification columns (ThermoFisher) following the manufacturer's protocol.

Transcription of mVenus and mCherry mRNA

In vitro transcription was performed using the linear DNA templates prepared, with the HiScribe™ T7 High Yield RNA Synthesis Kit (NEB), following the manufacturer's protocol. The reactions were diluted by 10-fold with water and incubated in the presence of DNase I (ThermoFisher) for 15 minutes at 37 °C, to remove the DNA template. The resulting RNA was then purified using the GeneJet RNA Clean-up and Concentration Kit (ThermoFisher) following the manufacturer's protocol and eluted in H_2O. Prior to use, mRNA was tested for size and purity against a commercial ssRNA size marker.

RNase H-mediated mRNA degradation

1 pmol of mVenus mRNA was incubated with 6 U of RNase H (recombinant *E. coli*, Takara) and 0.2 pmol of antisense oligonucleotide (ASO) in a buffer system containing 30 mM HEPES pH 7, 100 mM KCl, 20 mM $MgCl_2$ and 2 mM DTT. The samples were incubated at 37 °C for 1 hour. After incubation, RNA loading dye (NEB, B0363S) was added, the samples heated to 70 °C for 10 minutes and analysed by agarose gel electrophoresis.

UV irradiation conditions

Samples in an open 200 µL PCR tube were held in a PCR tube rack (StarLabs) over aluminium foil and irradiated top-down. Irradiation was performed with a ThorLabs 365 nm LED (M365L3) equipped with a collimator (COP5-A) from a distance of 34 cm at an irradiance of 2.12 $mW \cdot cm^{-2}$, controlled by a ThorLabs driver (LEDD1B) set at 1 A maximum drive current. The power was measured at the position of the sample using the ThorLabs analog handheld laser power meter console PM100A, using a photodiode power sensor (Thorlabs S120VC), which has a measuring surface area of 0.7088 cm^2 (Ø of aperture: 0.95 cm).

Blue light irradiation conditions

Samples in an open 200 µL PCR tube were held in a PCR tube rack (StarLabs) over aluminium foil and irradiated top-down. Irradiation was performed with a ThorLabs 455 nm LED (M455L4) equipped with a collimator (COP4-A) from a distance of 30 cm at an irradiance of 64 $mW \cdot cm^{-2}$ for 1 minute. The power was measured at the position of the sample using the ThorLabs analog handheld laser power meter console PM100A, using a photodiode power sensor (Thorlabs S120VC), which has a measuring surface area of 0.7088 cm^2 (Ø of aperture: 0.95 cm).

In vitro transcription control using uvLA-V3

Reagents from the HiScribe™ T7 High Yield RNA Synthesis kit (NEB) were combined in a PCR tube as a master mix considering 3 µL final volume per reaction condition. Each 3 µL reaction contained the following: 0.05 µL T7 RNA Polymerase, 1X T7 RNA Polymerase buffer, and 10 mM NTPs, to which 0.001 pmol of mVenus linear DNA template, 3 U of RNase H (recombinant *E. coli*, Takara) and 0.05 pmol of caged ASO uvLA-**V3** were also added. The

reactions were incubated in the dark at 37 °C for 3 hours. At this point, a 3 µL aliquot was transferred to a different tube and irradiated with UV light (conditions above) for 5 minutes, while 0.05 pmol of amino-ASO **V3** were added to a separate 3 µL aliquot as control, before all tubes were put back at 37 °C and incubated for 1 more hour. Each reaction was finally quenched with 0.5 µL of 100 mM EDTA, RNA loading dye (NEB, B0363S) was added, the samples heated to 70 °C for 10 minutes and analysed by agarose gel electrophoresis.

Two-wavelength control of in vitro transcription

Reagents from the HiScribe™ T7 High Yield RNA Synthesis kit (NEB) were combined in a PCR tube as a master mix considering 3 µL final volume per reaction condition. Each 3 µL reaction contained the following: 0.05 µL T7 RNA Polymerase, 1X T7 RNA Polymerase buffer, 10 mM NTPs, to which 0.001 pmol of bLA-mVenus DNA, 3 U of RNase H (recombinant *E. Coli*, Takara) and 0.05 pmol of caged ASO uvLA-**V3** were also added. The reaction was started by irradiating the tube with blue light (conditions above) for 1 minute, followed by incubation in the dark at 37 °C. Every hour 3 µL were removed from the master mix reaction tube and quenched with 0.5 µL of 100 mM EDTA. After 3 hours, one 3 µL was separately irradiated with UV light (conditions above) for 5 minutes, then put back at 37 °C. All reactions were quenched with 0.5 µL of 100 mM EDTA after 1 more hour (4 hours total). The reaction controls included one reaction where no light was applied, and one reaction where only UV light was applied at the start. In parallel, an equivalent set of reactions were run with non-caged nucleic acids as control. A linear mV DNA template was added to start the reaction and the amino-ASO **V3** was added to initiate RNase H-mediated degradation. All reactions were finally mixed with RNA loading dye (NEB, B0363S), heated to 70 °C for 10 minutes and analysed by agarose gel electrophoresis.

Light-controlled gene knockdown in cell-free protein synthesis with uvLA-V3

In a 200 µL PCR tube, 5 ng/µl linear mV template DNA was added to PURExpress® (NEB, E6800) with 0.67 U/µL RNase H (Takara) and 0.2 ng/µL NH_2-**V3** or uvLA-**V3** ASO. The resulting solutions were kept at room temperature in the dark and illuminated with UV as required (5 minutes), before placing them in a thermocycler and incubated at 37 °C for 4 hours. 2 µL of each solution was then placed into 39 µL of H_2O and mixed by pipetting. 40 µL of the resulting solutions were then transferred into a 384 well plate and placed into a plate reader (Tecan Infinity M1000) and fluorescence measurements were taken ($\lambda_{Ex/Em}$: 515/527 nm, Gain 173).

Control of transcription and gene knockdown with two wavelengths in cell-free protein synthesis

In a 200 µL PCR tube, 5 ng/ul of blue light-activatable mV template DNA (as prepared previously)[1] were added to PURExpress® (NEB, E6800) with 0.67 U/µL RNase H (Takara) and 0.33 ng/µL uvLA-**V3** and 0.2 ng/µL NH_2-**V3** or uvLA-**V3** ASO. The resulting solutions were kept at room temperature in the dark and illuminated as required (1 minute with blue and 3 mins with UV), before placing them in a thermocycler and incubated at 37 °C. To illuminate at different timepoints, tubes were cooled to room temperature in the dark and illuminated as required, before placing them back into the thermocycler for a total of 4 hours incubation time. 2 µL of each solution were then placed into 39 µL of H_2O and mixed by pipetting. 40 µL of the resulting solutions were then transferred into a 384 well plate and placed into a plate reader

(Tecan Infinity M1000) and fluorescence measurements were taken ($\lambda_{Ex/Em}$: 515/527 nm, Gain 173).

Two-wavelength control of RNase H-mediated mRNA degradation

1 pmol of either mV or mC mRNA was incubated with 6 U of RNase H (recombinant *E. coli*, Takara) and 1.2 ng of each bLA-**C1** and uvLA-**V3** in a buffer system containing 30 mM HEPES pH 7, 100 mM KCl, 20 mM $MgCl_2$ and 2 mM DTT. The samples were illuminated as required (5 minutes for UV irradiation or 1 minute for blue irradiation), incubated at 37 °C for 1 hour. After incubation, RNA loading dye (NEB, B0363S) was added, the samples heated to 70 °C for 10 minutes and analysed by agarose gel electrophoresis.

Two wavelength-controlled gene knockdown in cell-free protein synthesis

In a 200 µL PCR tube, 5 ng/µl each of linear mV and mC DNA (as prepared were added to PURExpress® (NEB, E6800) with 0.67 U/µL RNase H (Takara), 0.5 ng/µL bLA-**C1** ASO and 0.33 ng/µL uvLA-**V2** ASO. The resulting solutions were kept at room temperature in the dark and illuminated as required (3 minutes for UV irradiation or 1 minute for blue irradiation), before placing them in a thermocycler at 37 °C for 4 hours. 2 µL of each solution were then placed into 39 µL of H_2O and mixed by pipetting. 40 µL of the resulting solutions were then transferred into a 384 well plate and placed into a plate reader (Tecan Infinity M1000) and fluorescence measurements were taken ($\lambda_{Ex/Em}$: 515/527 nm for mV, gain 183, and $\lambda_{Ex/Em}$: 587/610 nm for mCherry, gain 234).

Agarose gel electrophoresis

Agarose gels were prepared at 1.5% agarose in 1X TBE buffer and 1X Gel-Red® nucleic acid stain (Biotium), then run at 100 V in 1X TBE buffer. Samples were prepared using RNA loading dye (NEB, B0363S), heated to 70 °C for 10 minutes then cooled on ice before loading to denature the RNA. Samples were run against a low-range ssRNA ladder (NEB, N0364S) or ssRNA ladder (NEB, N0362S).

RNase H-mediated degradation of mRNA experiments

RNase H-mediated mVenus-mRNA degradation controlled by UV

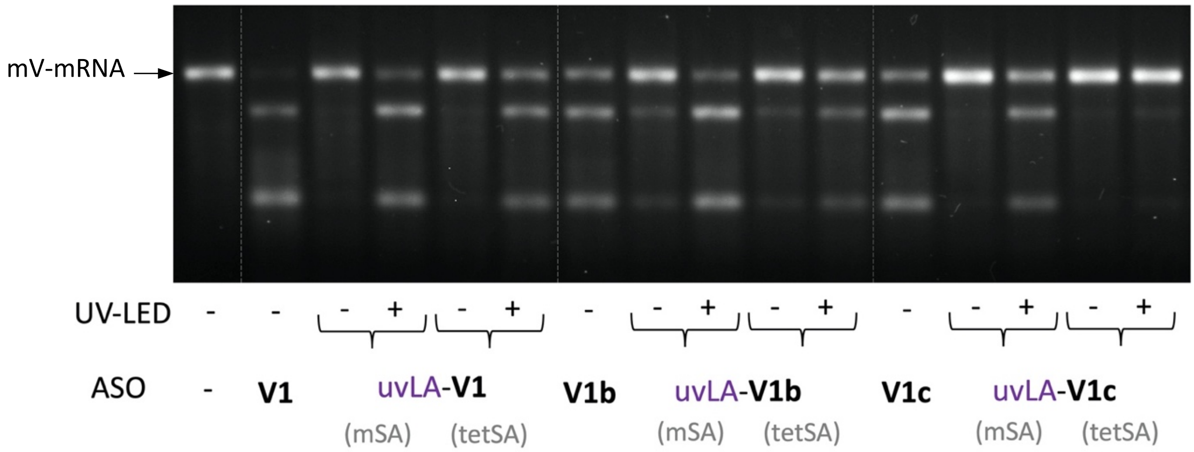

Supplementary Figure 1. Agarose gel showing RNase H-mediated mRNA degradation to compare the activity of **V1** after modification in different positions. The amino-modified antisense oligonucleotide (ASO) **V1** is the most active compared to **V1b** and **V1c**. The activity before and after UV irradiation of the three ASOs modified with uvLA-biotin and binding of monovalent (mSA) or tetravalent streptavidin (tetSA) is also compared. uvLA-**V1** and uvLA-**V1c** show a much better on/off ratio compared to uvLA-**V1b**, with uvLA-**V1** showing better mRNA degradation after UV. Before UV irradiation, binding of mSA or tetSA completely prevents RNase H activity, whereas after applying UV, the photocleavable group containing mSA cleaves off more easily than with tetSA.

RNase H-mediated mVenus-mRNA degradation with different unmodified ASO sequences

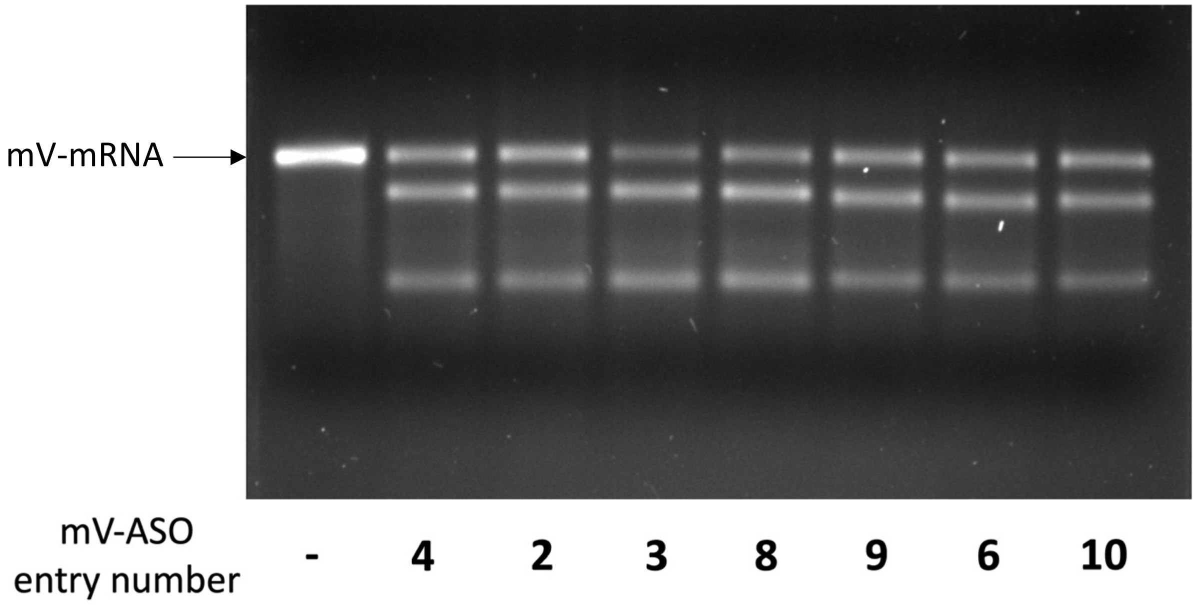

Supplementary Figure 2. Agarose gel showing RNase H-mediated mRNA degradation to compare the activity of different ASO sequences. All ASOs shown have a similar ability to degrade mVenus-mRNA. For the following experiments ASO entry **6** was selected and modified. From Supplementary Table 4: ASO entry **4** = **V1c**, ASO entry **2** = **V1**, ASO entry **3** = **V1b**.

Control of in vitro transcription experiments

In vitro transcription of mVenus-DNA controlled with UV light

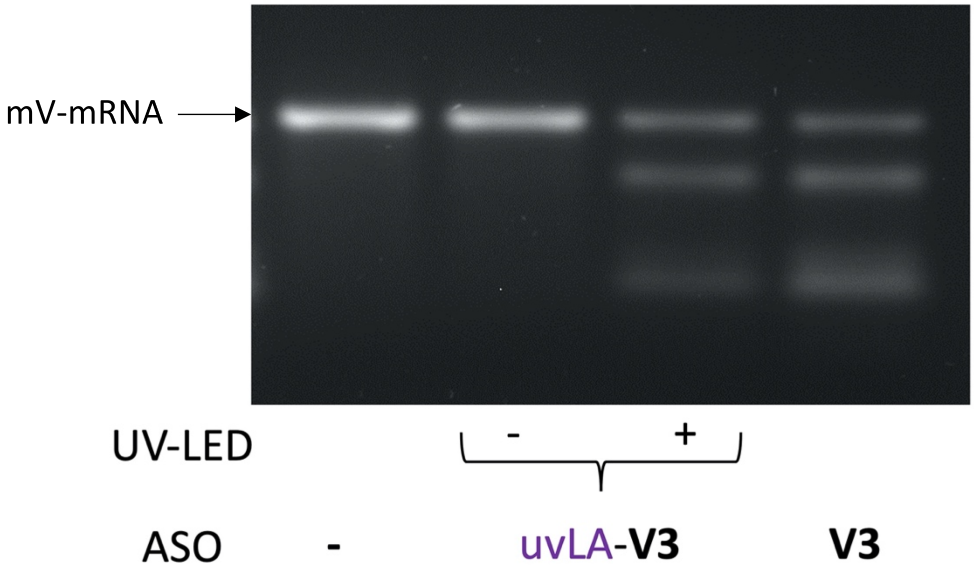

Supplementary Figure 3. Agarose gel showing light-controlled in vitro transcription, after four hours of incubation. From the left: prior UV irradiation, the amount of mRNA formed is equal in the absence (lane 1) or presence (lane 2) of the photocaged ASO uvLA-**V3**. Once UV light is applied, the mRNA formed is degraded by the now uncaged uvLA-**V3** (lane 3). The degradation observed is at similar levels to the amino-ASO control **V3** (lane 4).

Two-wavelength control of in vitro transcription of mVenus

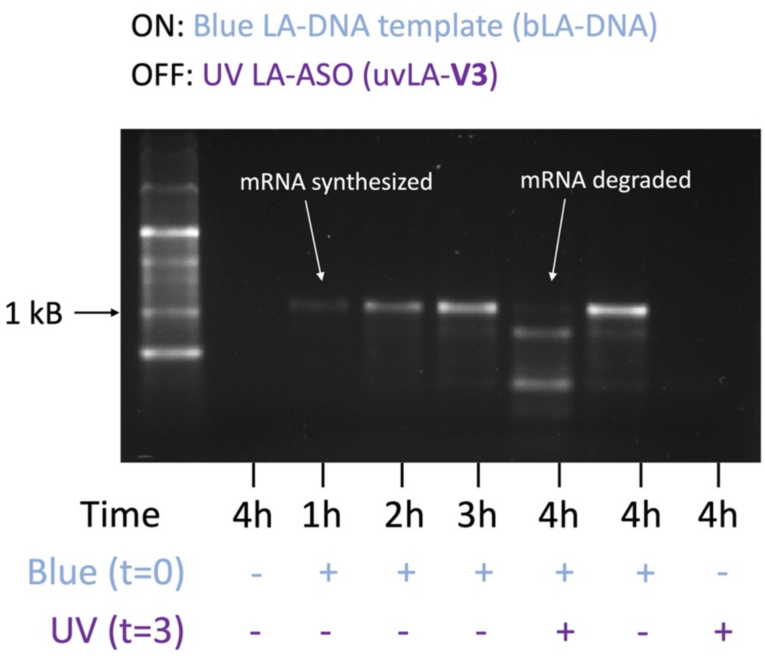

Supplementary Figure 4. In vitro transcription (in the presence of RNase H) controlled with two orthogonal wavelengths of light displayed on an agarose gel. The reaction is activated by uncaging bLA-DNA with blue light and mVenus-mRNA is produced for three hours. The mRNA formed can then be degraded by RNase H once UV light is applied to uncage uvLA-**V3**. The reaction does not occur if no light is applied. Irradiation with only UV light does not activate bLA-DNA, showing orthogonality between the two light sensitive groups.

Control of mVenus transcription with amino-ASO

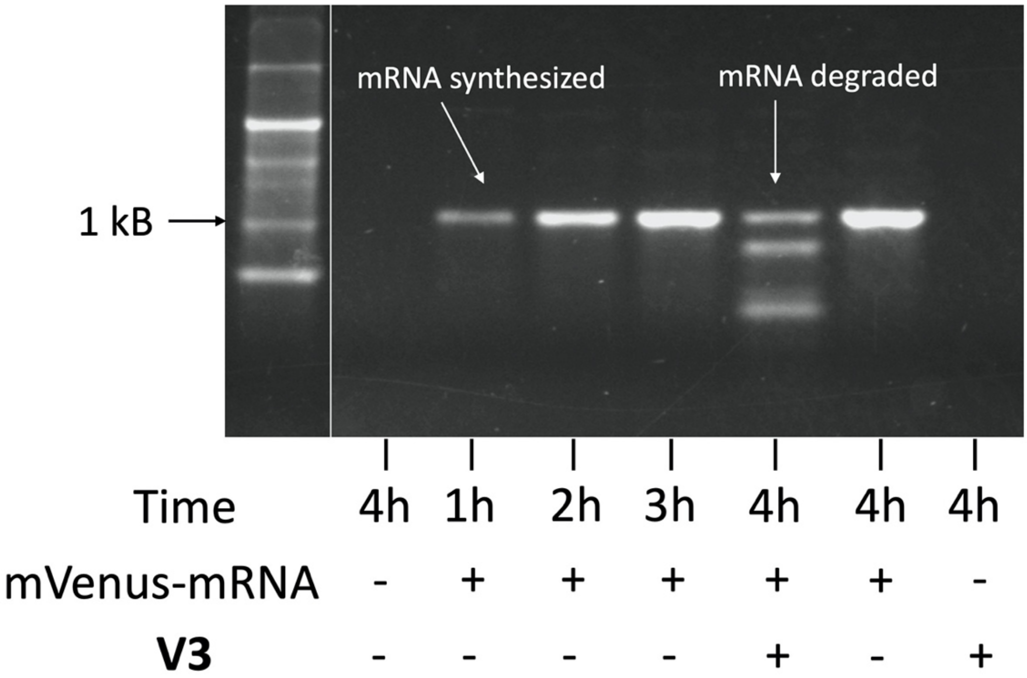

Supplementary Figure 5. In vitro transcription (IVT) reaction (in the presence of RNase H) carried out as a control to the bLA-DNA and uvLA-ASO experiment in Supplementary Figure 4. Purified mVenus-mRNA is added to the IVT mixture to start the reaction and mRNA is produced. After three hours, the amino-ASO **V3** is added to the reaction mixture and the mRNA formed is degraded by RNase H. All the steps correspond to the light activation steps shown in Supplementary Figure 4.

Initial screening of antisense oligonucleotides targeting mCherry

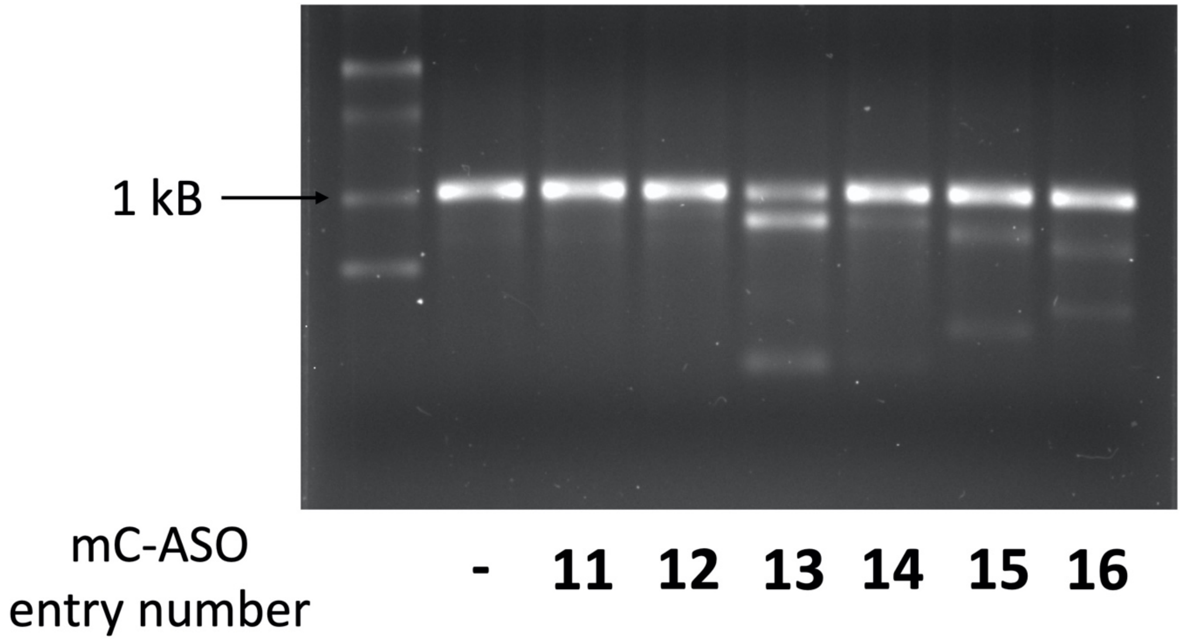

Supplementary Figure 6. Agarose gel showing RNase H-mediated mRNA degradation to compare the activity of different ASO sequences on mCherry. The numbers refer to the ASOs used from Supplementary Table 4. ASO entry **13** was selected after this experiment and its sequence optimized further.

Evaluation of mCherry-ASOs crosstalk to mVenus-mRNA

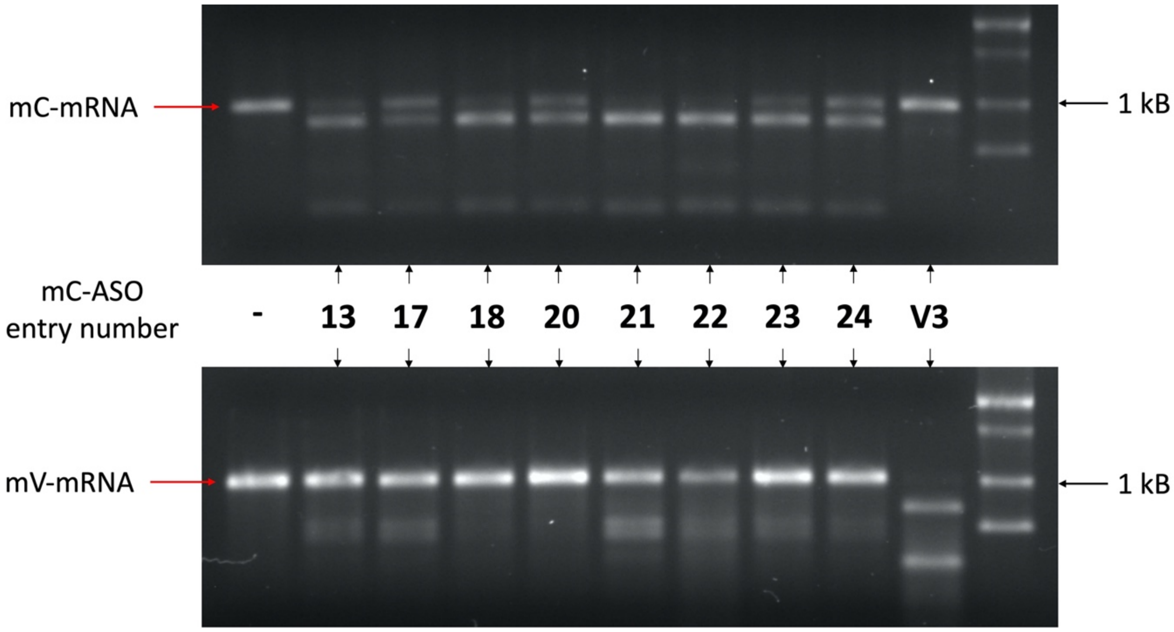

Supplementary Figure 7. Agarose gel showing RNase H-mediated mRNA degradation to compare the activity of different ASOs targeting mCherry-mRNA, with sequence length and area targeted on mRNA similar to those of ASO **13**. The sequences were also screened against mVenus-mRNA to evaluate the crosstalk. The numbers refer to the ASOs used from Supplementary Table 4. ASO sequence **18** showed the best activity against mCherry and lowest against mVenus, and it was therefore selected for further experiments.

Orthogonality between mCherry- and mVenus-ASOs

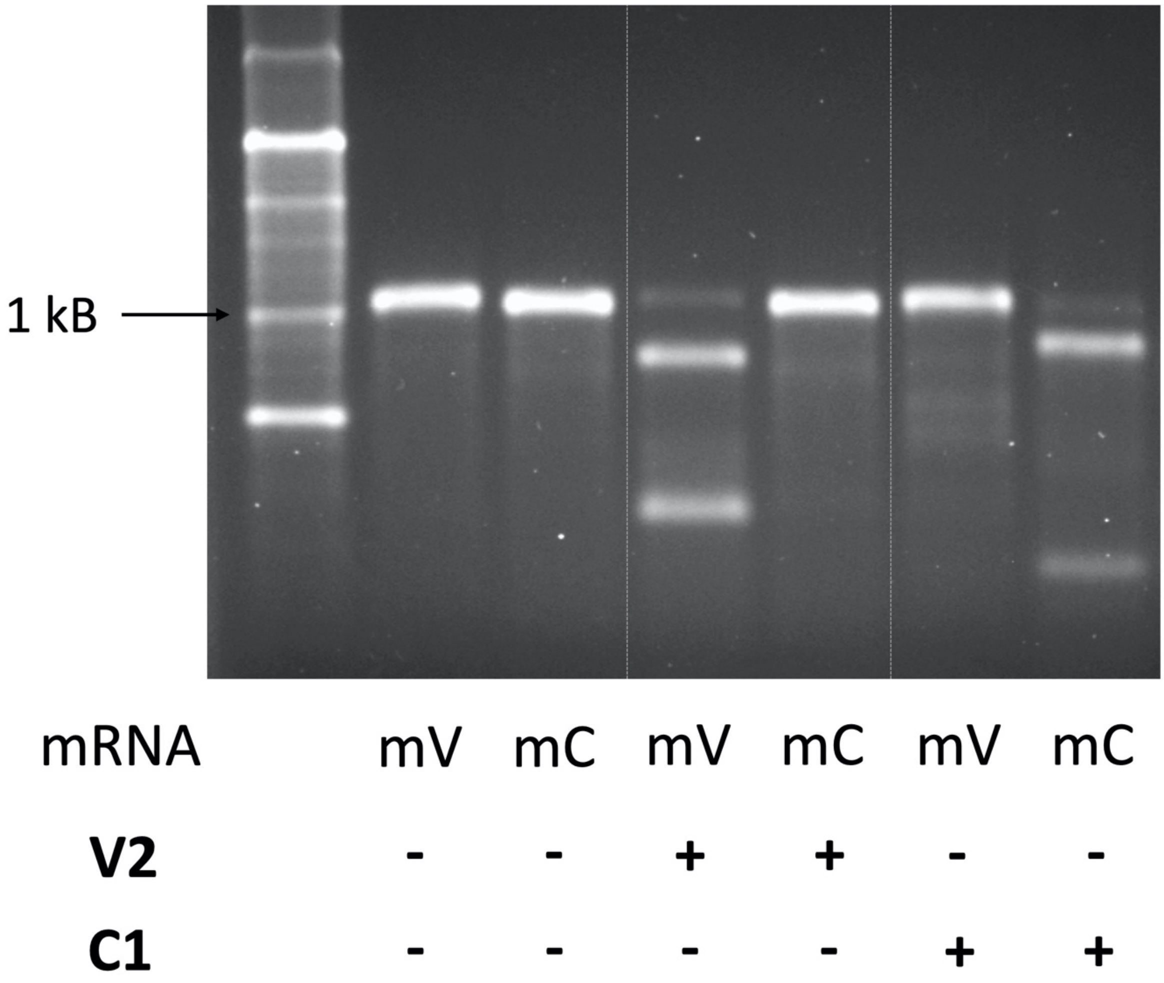

Supplementary Figure 8. Agarose gel showing RNase H-mediated mRNA degradation to evaluate the crosstalk between antisense oligonucleotides (ASOs) targeting different mRNA sequences. Each lane corresponds to a reaction containing purified mVenus-mRNA or mCherry-mRNA. As expected, the amino-ASO **V2** is only degrading mVenus-mRNA, whereas **C1** is only degrading mCherry-mRNA. **V3**, which was the most active ASO tested for mVenus was also tested against mCherry-mRNA (not shown), but it caused more mCherry-mRNA degradation than **V2**.

HPLC purification of biotinylated antisense oligonucleotides

Preparation of uvLA-V1 biotin

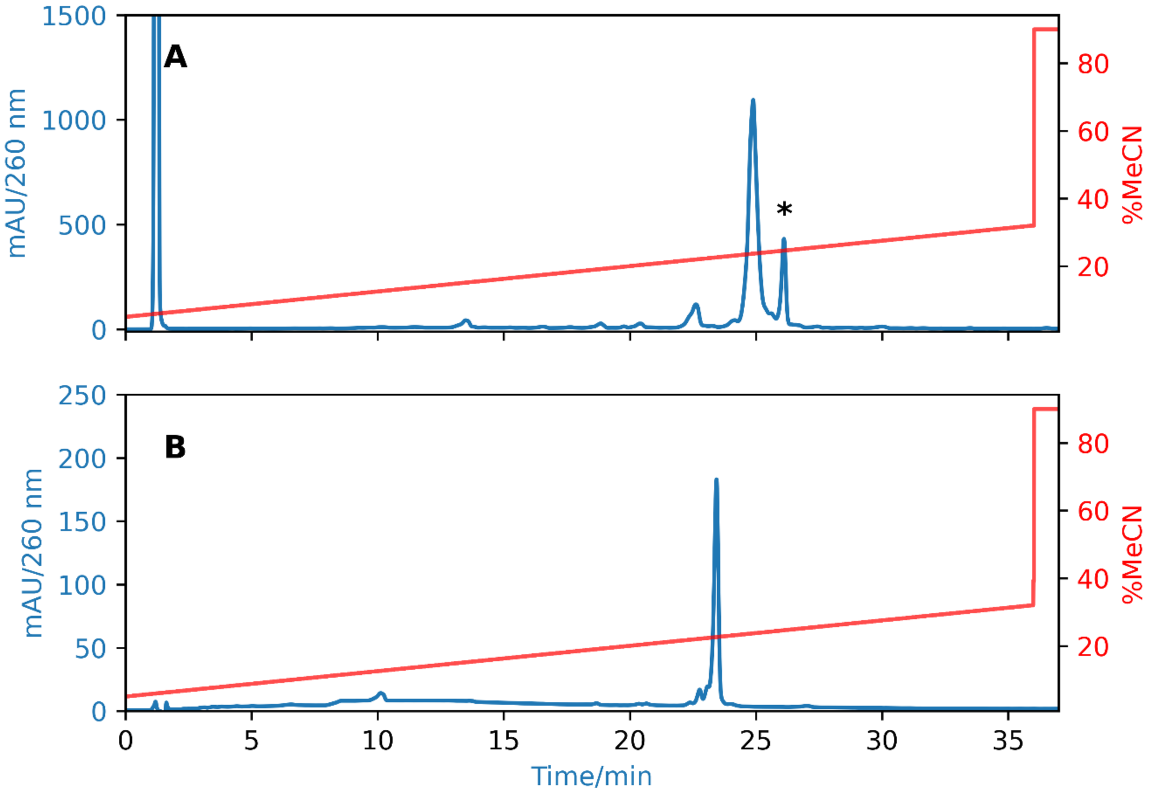

A) Crude HPLC trace of **C1** after reaction with bLA-biotin. The desired oligonucleotide is highlighted by " * ". **B**) HPLC trace after HPLC purification.

Preparation of uvLA-V2 biotin

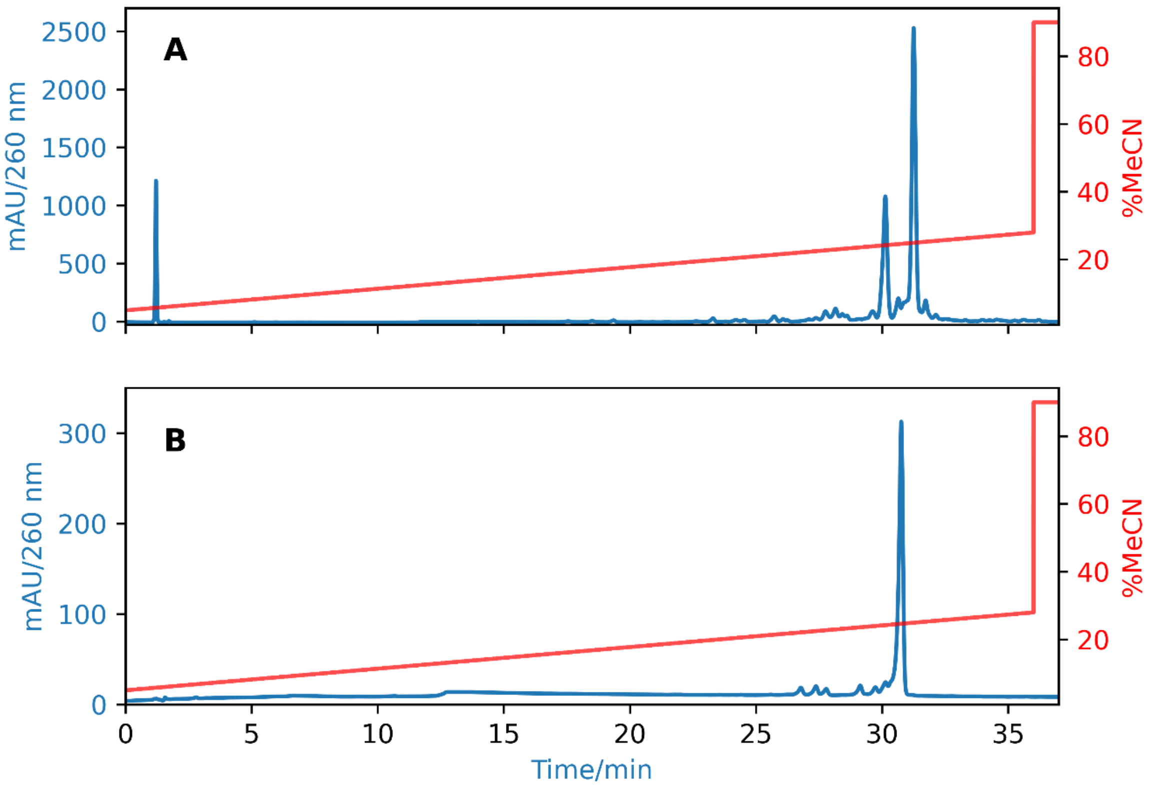

A) Crude HPLC trace of **V2** after reaction with uvLA-biotin. **B**) HPLC trace after HPLC purification.

Preparation of uvLA-V3 biotin

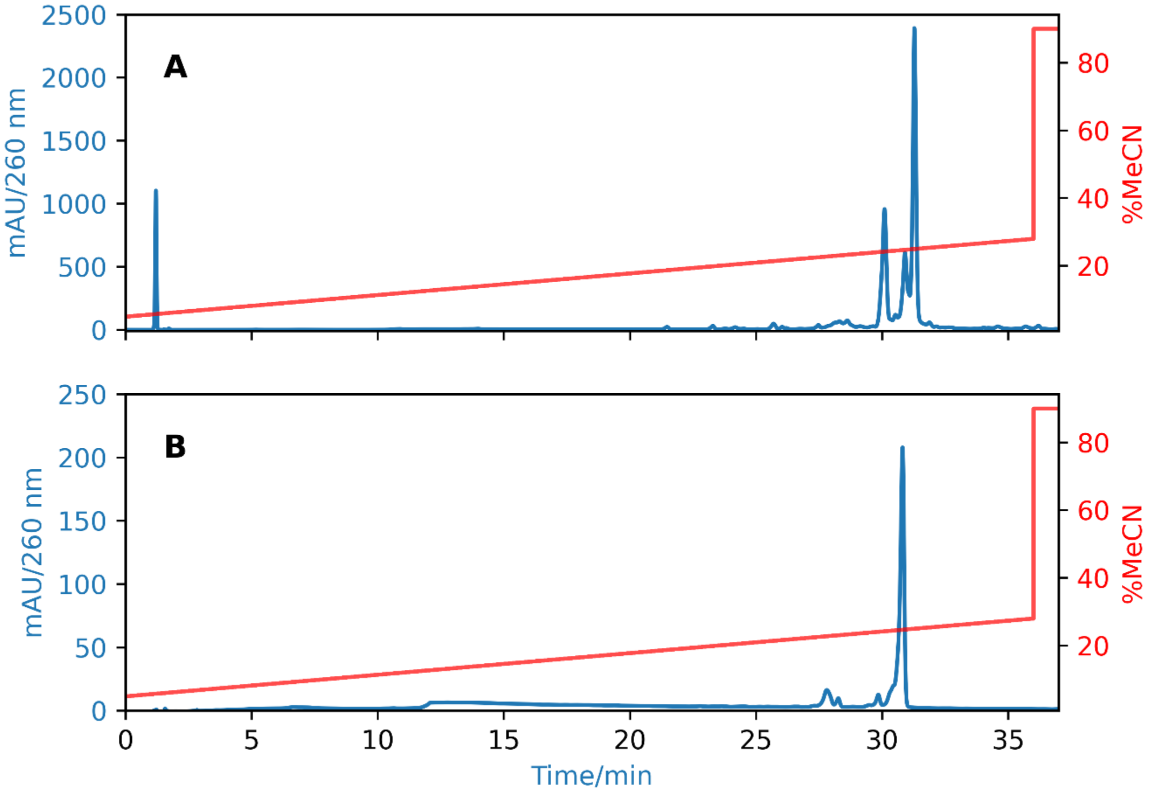

A) Crude HPLC trace of **V3** after reaction with uvLA-biotin. **B**) HPLC trace after HPLC purification.

Preparation of bLA-C1 biotin

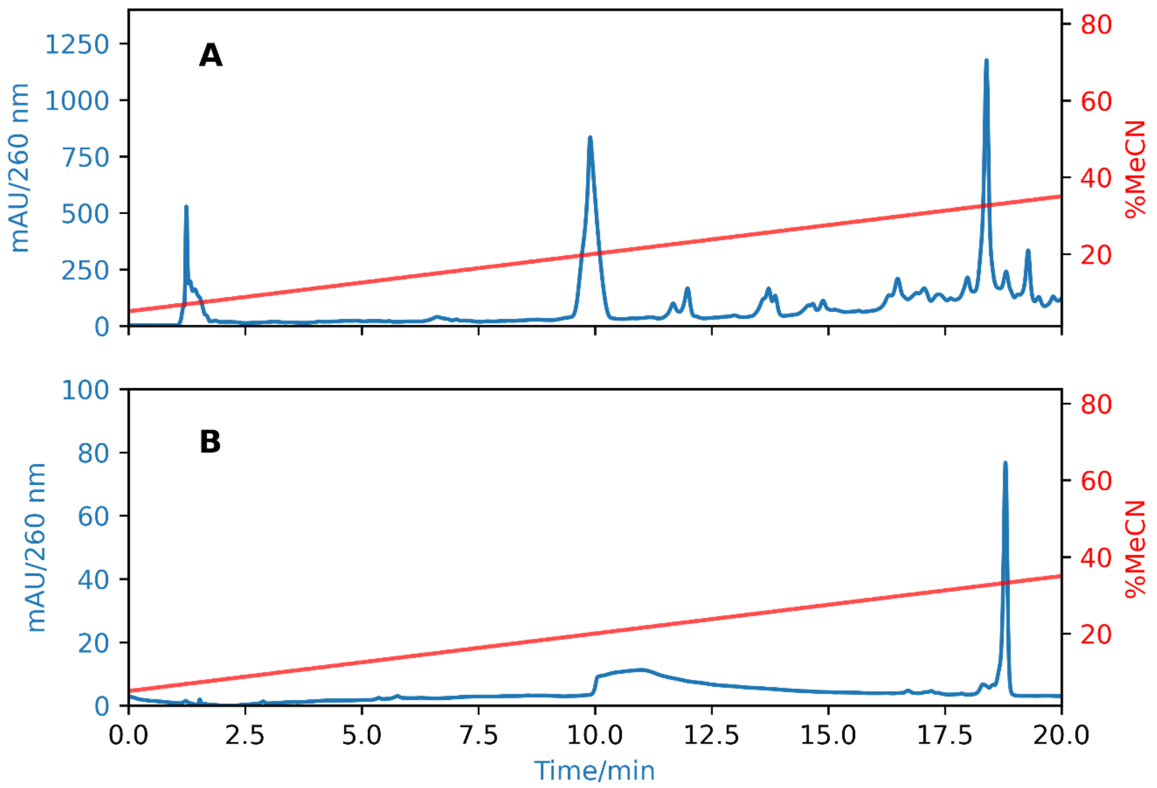

A) Crude HPLC trace of **C1** after reaction with bLA-biotin. **B**) HPLC trace after HPLC purification.

UV-Vis Traces of LA-ASOs

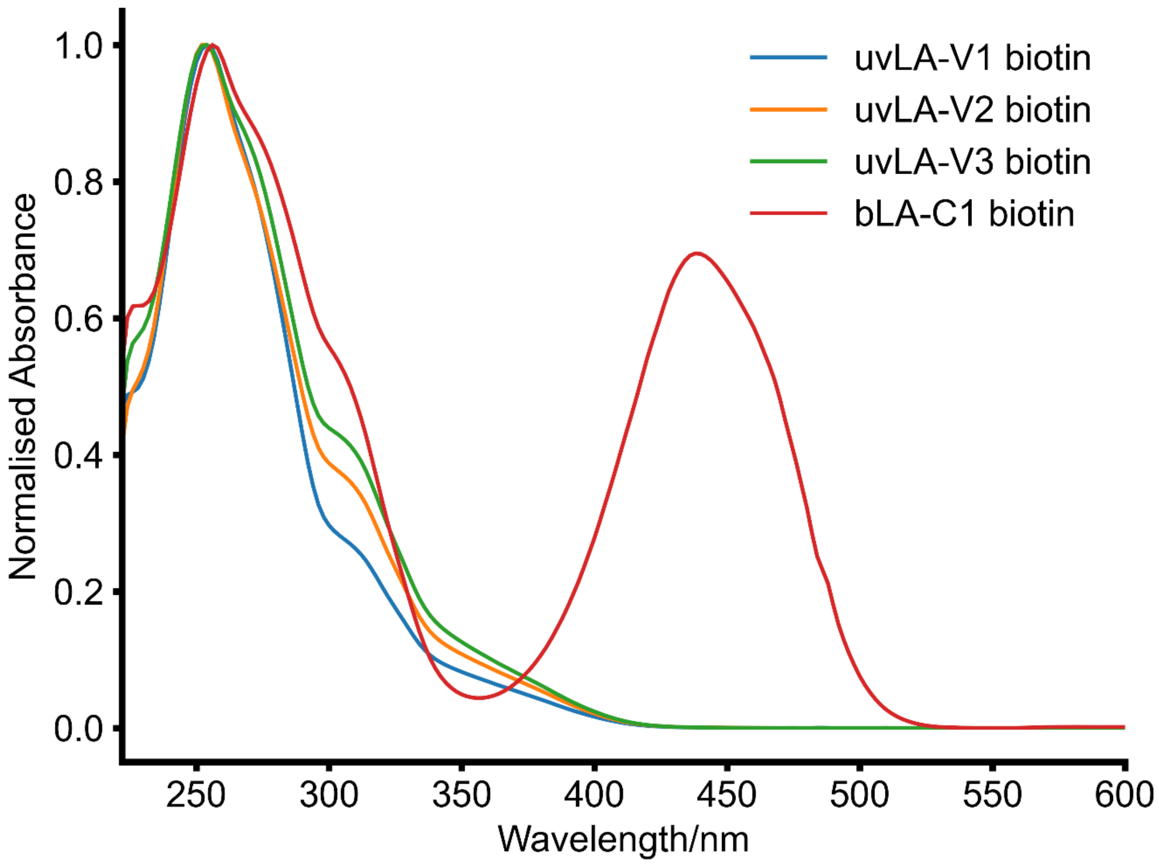

UV-Visible Absorbance traces of LA-ASOs as recorded by HPLC and normalised to λ_{max} at ~260nm.

Oligonucleotide Mass Spectrometry

Oligonucleotide Mass Spectra were recorded on a Waters Xevo G2 QTOF ESI- UPLC-MS system. A gradient of MeOH in Et_3N and hexafluoroisopropanol (HFIP) was used (buffer A, 8.6 mM Et_3N, 200 mM HFIP in 5% MeOH/H_2O (v/v); buffer B, 20% buffer A in MeOH). Samples were analysed using a gradient of 0 to 70% buffer B over 8 minutes and 0% B to 10 minutes and data was then deconvoluted using MassLynx v4.1.

**Entry**	**Oligonucleotide**	**Number of PC-biotin groups**	**Purified product**	**Expected mass**	**Mass after deconvolution**
1	**V1**	3	uvLA-**V1** biotin	9149	9148
2	**V2**	4	uvLA-**V2** biotin	9740	9740
3	**V3**	4	uvLA-**V3** biotin	9032	9032
4	**C1**	4	bLA-**C1** biotin	8320	8320

LC-MS spectra of uvLA-V1 biotin

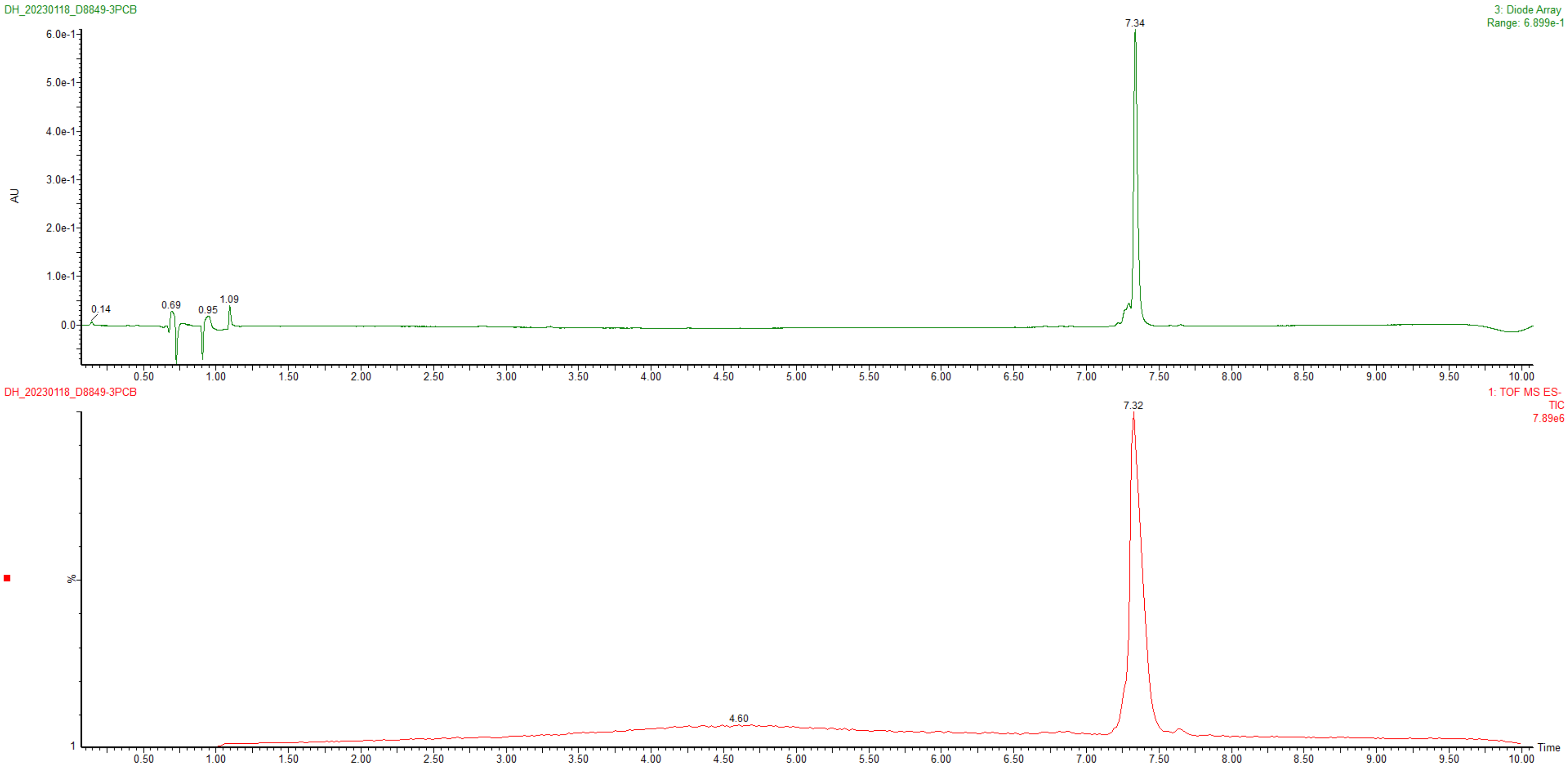

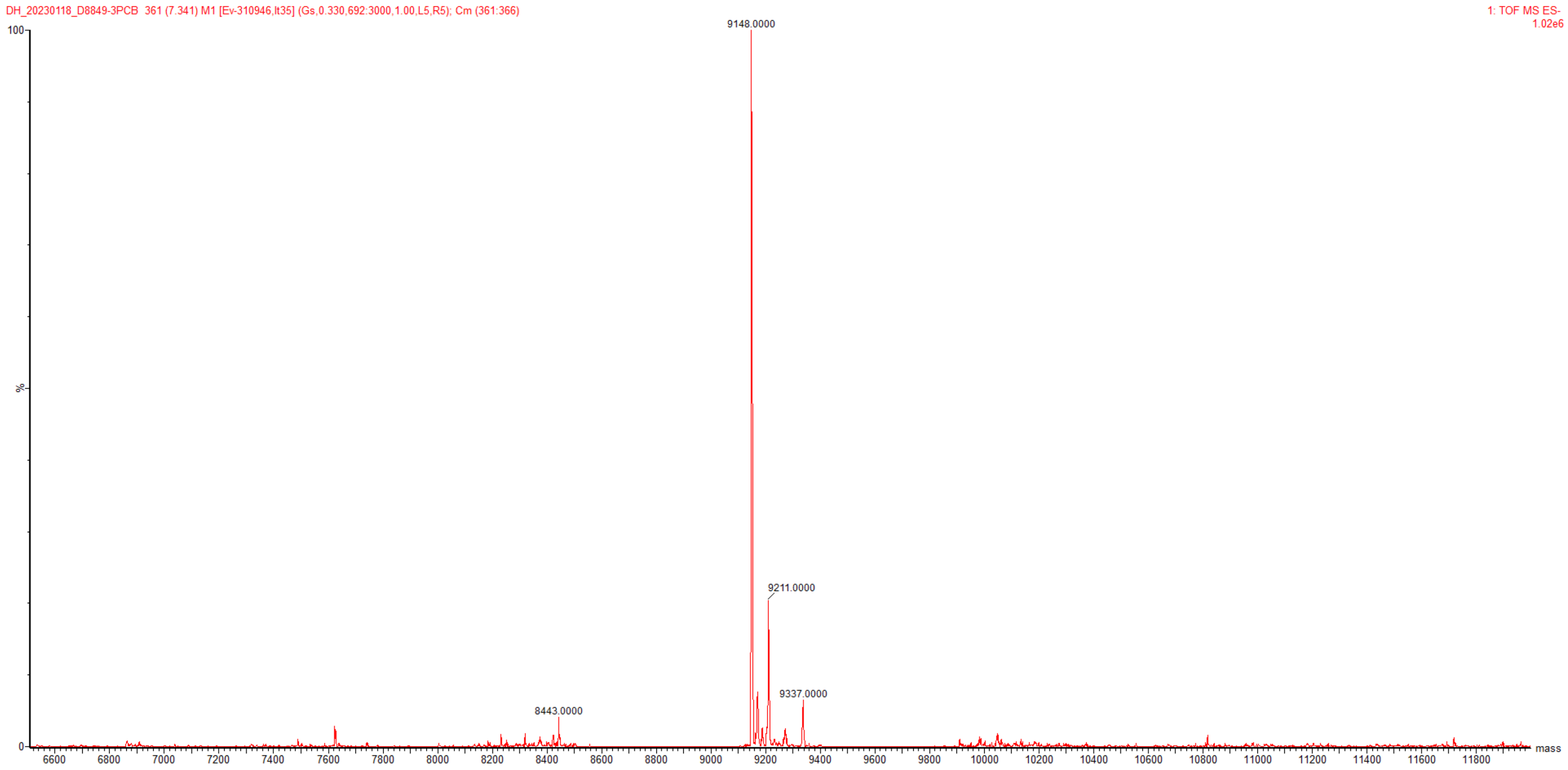
DH_20230118_D8849-3PCB 361 (7.341) M1 [Ev-310946,It35] (Gs,0.330,692:3000,1.00,L5,R5); Cm (361:366)
1: TOF MS ES-
1.02e6
9148.0000
9211.0000
9337.0000
8443.0000
100
%
0
6600
6800
7000
7200
7400
7600
7800
8000
8200
8400
8600
8800
9000
9200
9400
9600
9800
10000
10200
10400
10600
10800
11000
11200
11400
11600
11800
mass

LC-MS spectra of uvLA-V2 biotin

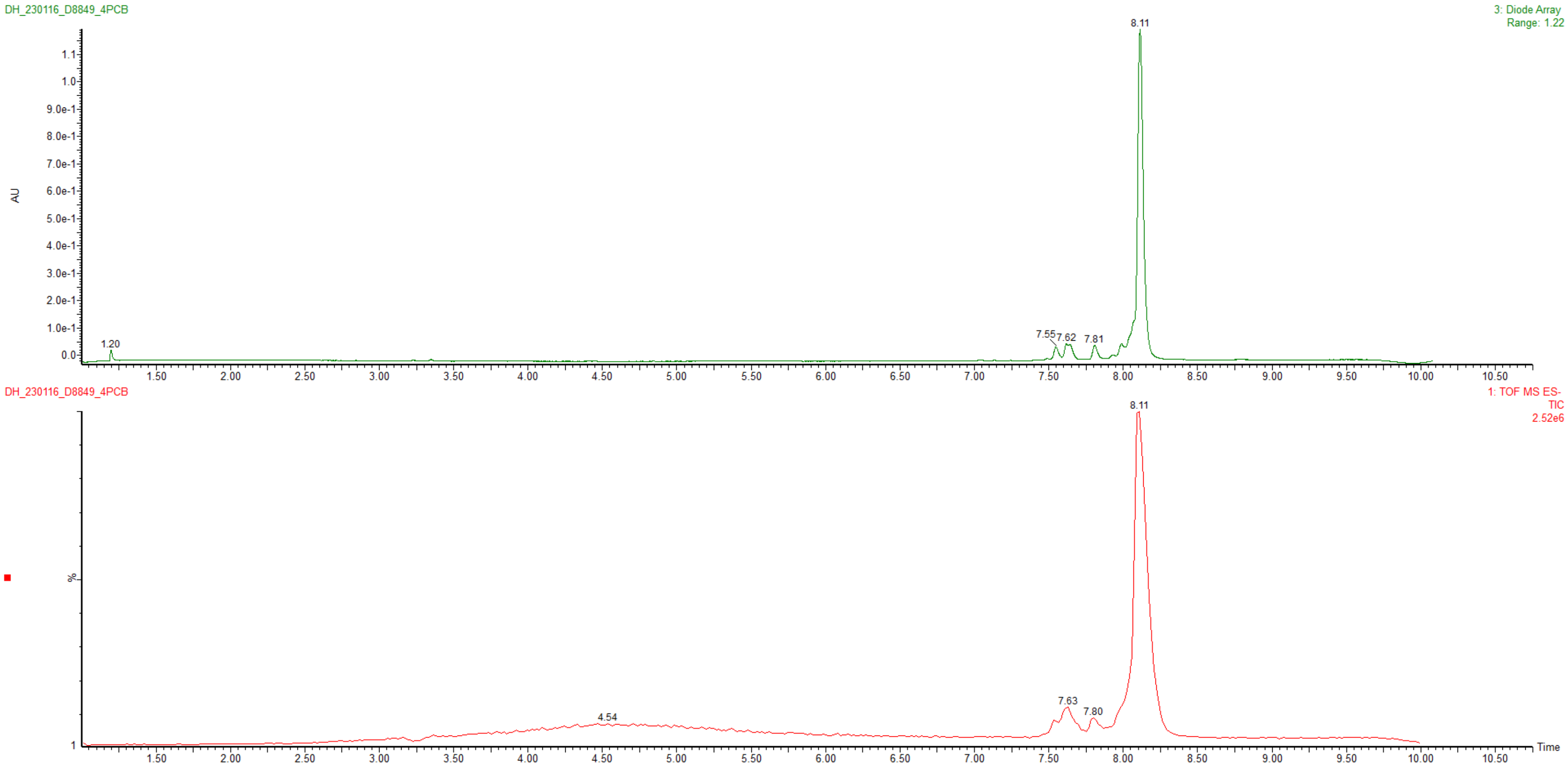

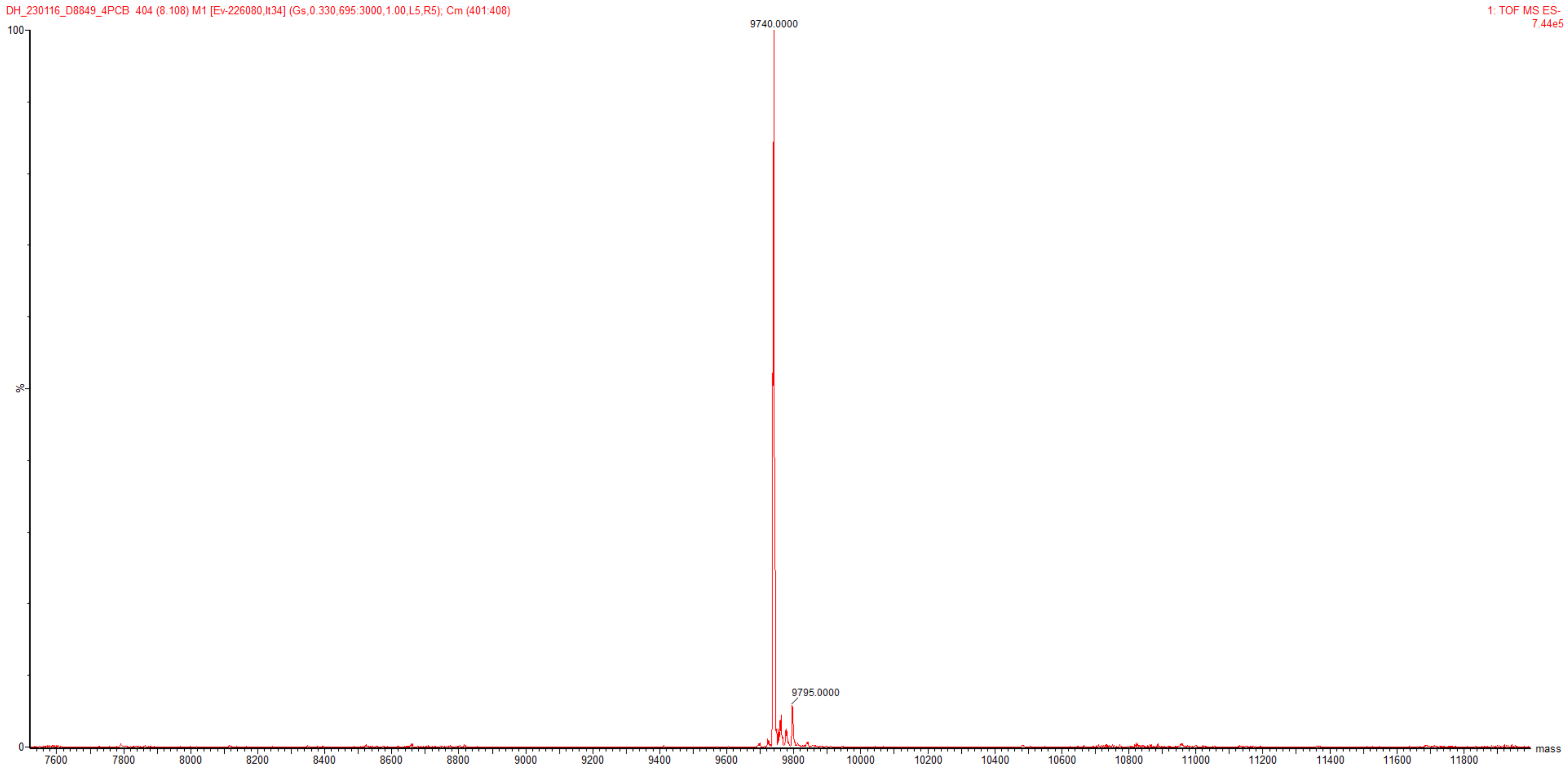
DH_230116_D8849_4PCB 404 (8.108) M1 [Ev-226080,It34] (Gs,0.330,695:3000,1.00,L5,R5); Cm (401:408)
1: TOF MS ES-
7.44e5
9740.0000
9795.0000
100
%
0
7600
7800
8000
8200
8400
8600
8800
9000
9200
9400
9600
9800
10000
10200
10400
10600
10800
11000
11200
11400
11600
11800
mass

LC-MS spectra of uvLA-V3 biotin

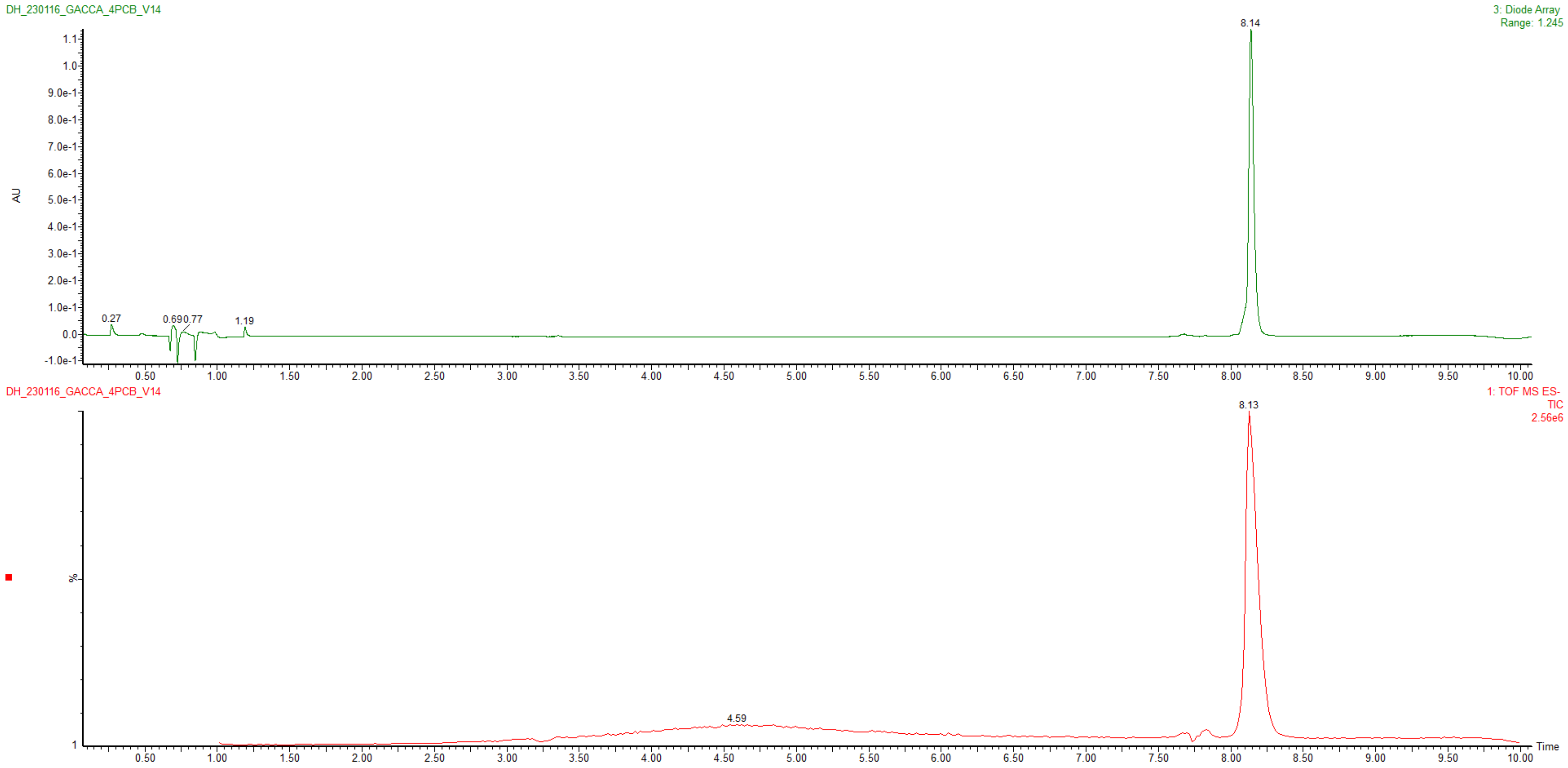

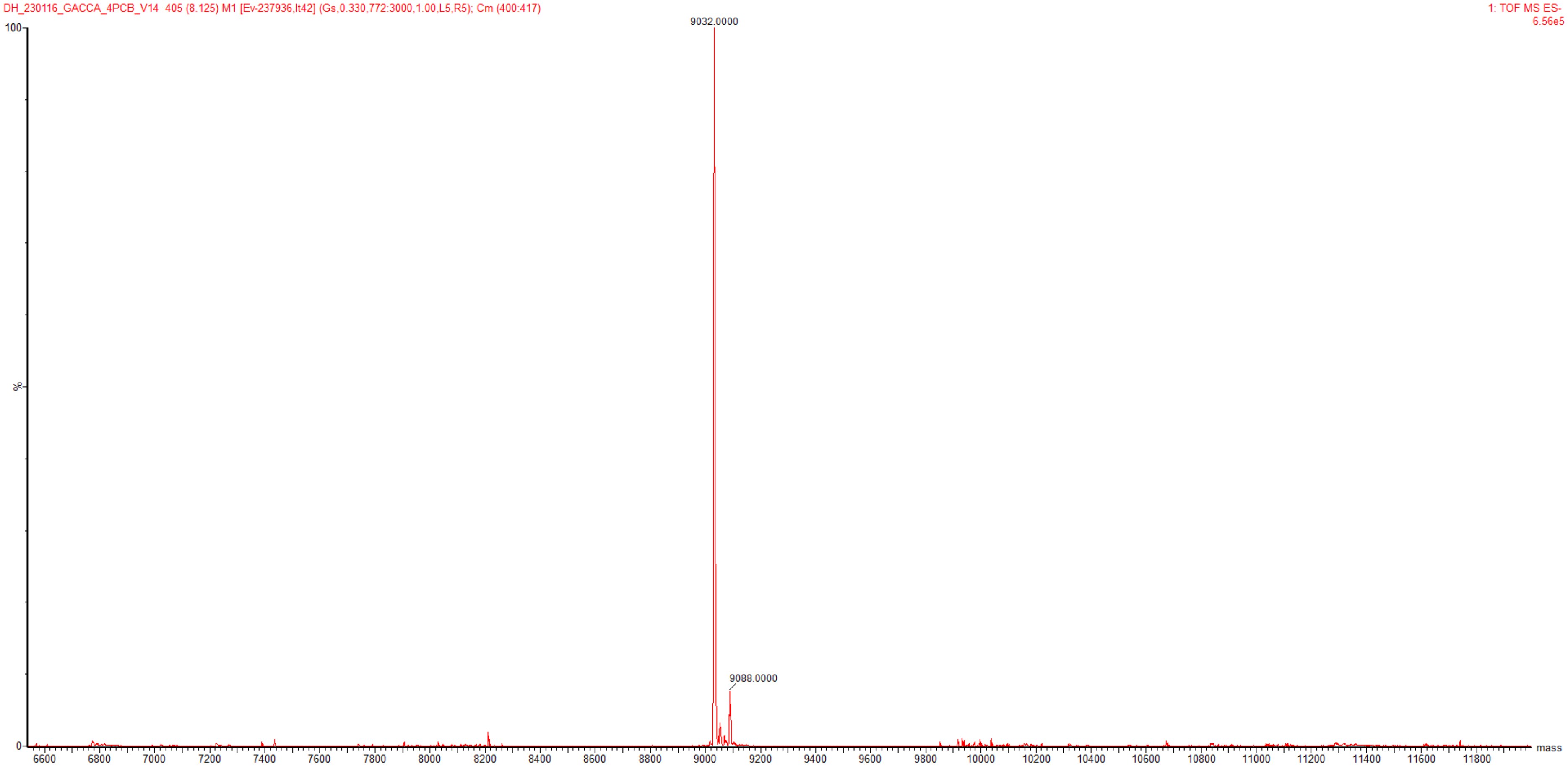
DH_230116_GACCA_4PCB_V14 405 (8.125) M1 [Ev-237936,It42] (Gs,0.330,772:3000,1.00,L5,R5); Cm (400:417)
1: TOF MS ES-
6.56e5
9032.0000
9088.0000
100
%
0
mass
6600
6800
7000
7200
7400
7600
7800
8000
8200
8400
8600
8800
9000
9200
9400
9600
9800
10000
10200
10400
10600
10800
11000
11200
11400
11600
11800

LC-MS Spectra of bLA-C1 biotin

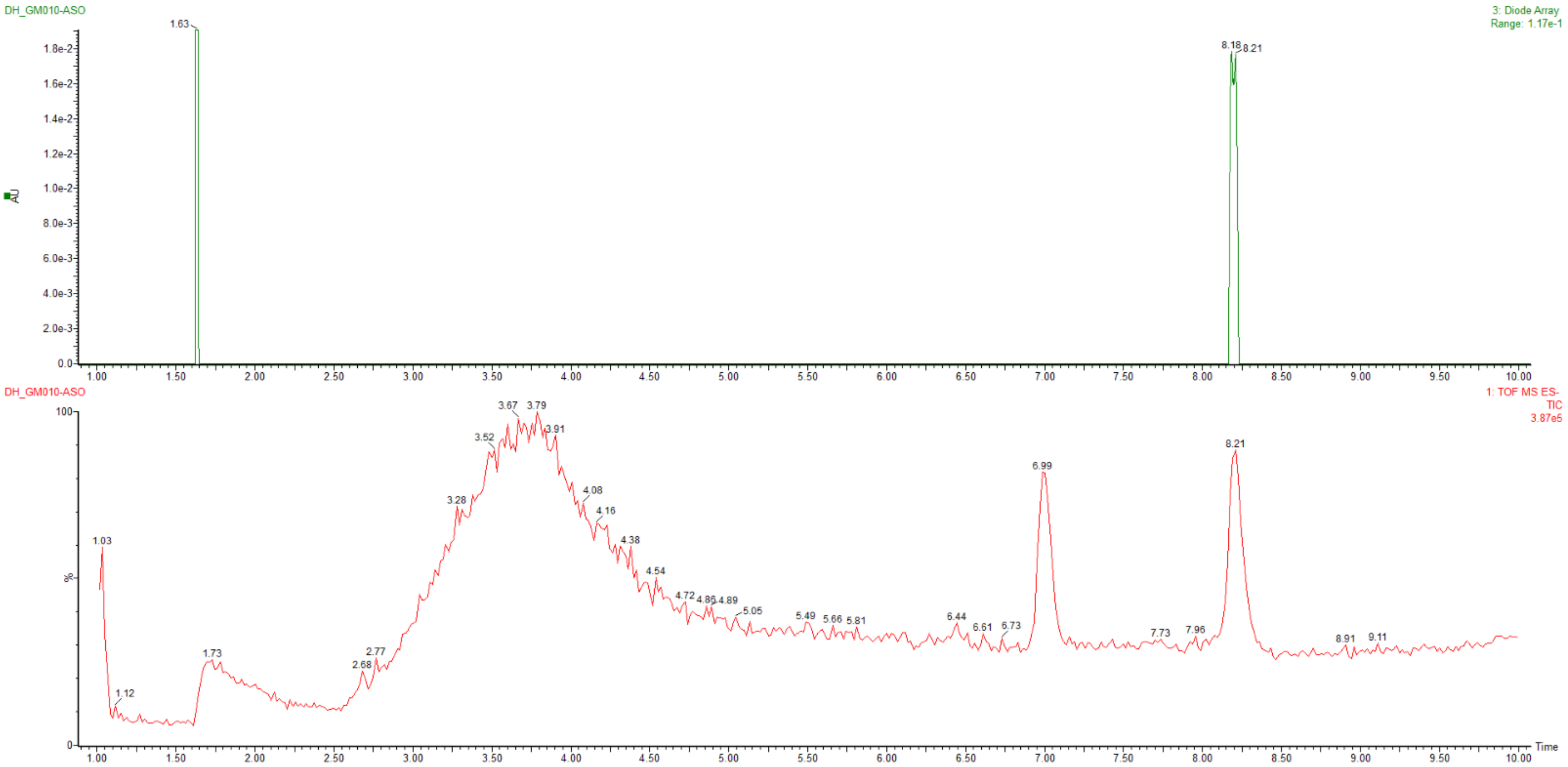

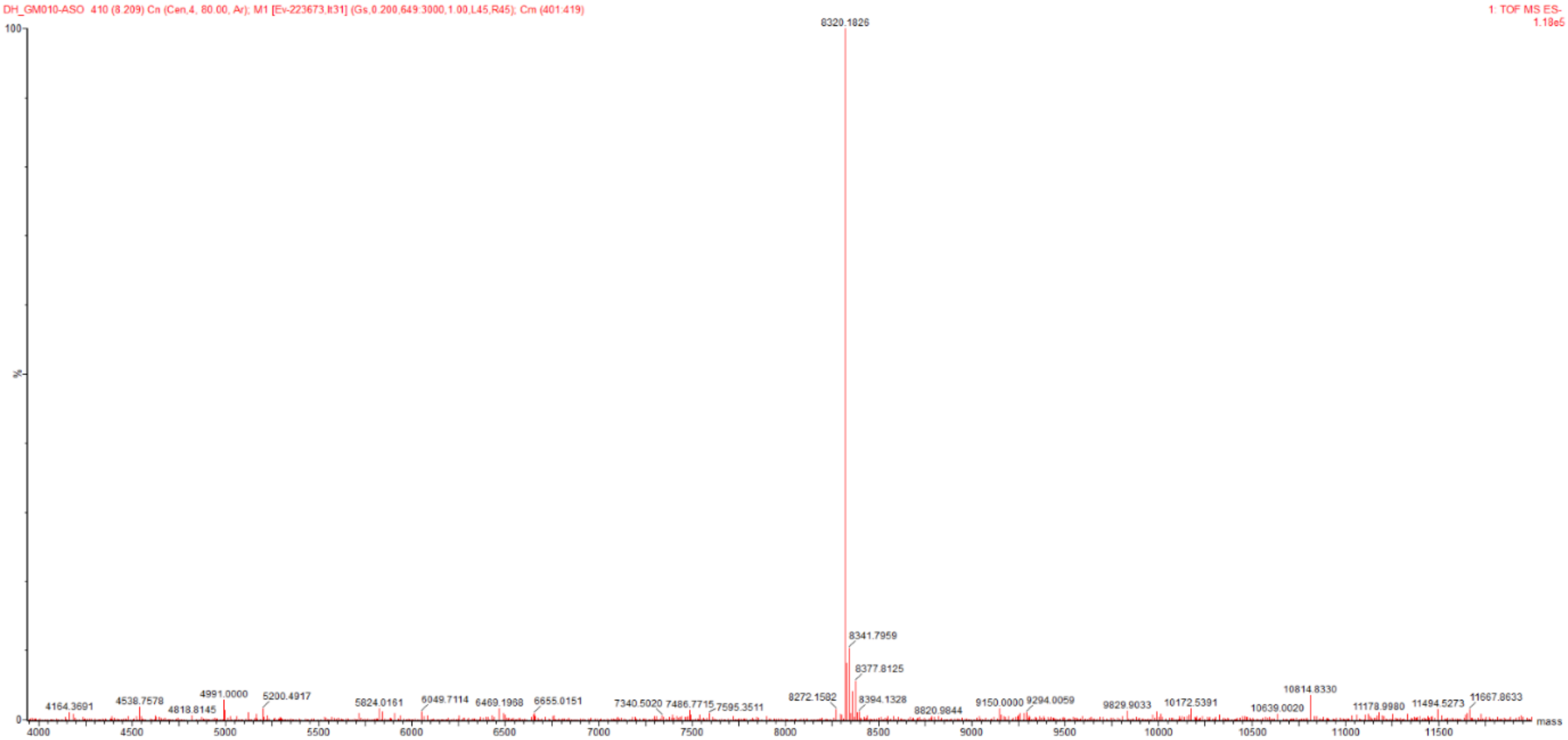

DH_GM010-ASO 410 (8.209) Cn (Cen,4, 80.00, Ar); M1 [Ev-223673,It31] (Gs,0.200,649:3000,1.00,L45,R45); Cm (401:419)
1: TOF MS ES-
1.18e5
8320.1826
8341.7959
8377.8125
8394.1328
8272.1582
4164.3691
4538.7578
4818.8145
4991.0000
5200.4917
5824.0161
6049.7114
6469.1968
6655.0151
7340.5020
7486.7715
7595.3511
8820.9844
9150.0000
9294.0059
9829.9033
10172.5391
10639.0020
10814.8330
11178.9980
11494.5273
11667.8633
%
mass

REFERENCES

(1) Hartmann, D.; Chowdhry, R.; Smith, J. M.; Booth, M. J. Blue Light-Activatable DNA for Remote Control of Cell-Free Logic Gates and Synthetic Cells. *ChemRxiv* **2022**. https://doi.org/10.26434/chemrxiv-2022-p8xgb.
